# Anatomically plausible segmentations: Explicitly preserving topology through prior deformations

**DOI:** 10.1016/j.media.2024.103222

**Published:** 2024-06-15

**Authors:** Madeleine K. Wyburd, Nicola K. Dinsdale, Mark Jenkinson, Ana I.L. Namburete

**Affiliations:** a Oxford Machine Learning Neuroimaging Lab (OMNI) Computer Science Department, University of Oxford, Oxford, OX1 3QG, United Kingdom; b Wellcome Centre for Integrative Neuroimaging, Wellcome Centre for Integrative Neuroimaging, Oxford, United Kingdom; c Australian Institute for Machine Learning (AIML), Department of Computer Science, University of Adelaide, Adelaide, Australia; d South Australian Health and Medical Research Institute (SAHMRI), North Terrace, Australia

**Keywords:** Topology, Segmentation, Topology-preserving fields, Spatial transformer network

## Abstract

Since the rise of deep learning, new medical segmentation methods have rapidly been proposed with extremely promising results, often reporting marginal improvements on the previous state-of-the-art (SOTA) method. However, on visual inspection errors are often revealed, such as topological mistakes (e.g. holes or folds), that are not detected using traditional evaluation metrics. Incorrect topology can often lead to errors in clinically required downstream image processing tasks. Therefore, there is a need for new methods to focus on ensuring segmentations are topologically correct. In this work, we present TEDS-Net: a segmentation network that preserves anatomical topology whilst maintaining segmentation performance that is competitive with SOTA baselines. Further, we show how current SOTA segmentation methods can introduce problematic topological errors. TEDS-Net achieves anatomically plausible segmentation by using learnt topology-preserving fields to deform a prior. Traditionally, topology-preserving fields are described in the continuous domain and begin to break down when working in the discrete domain. Here, we introduce additional modifications that more strictly enforce topology preservation. We illustrate our method on an open-source medical heart dataset, performing both single and multi-structure segmentation, and show that the generated fields contain no folding voxels, which corresponds to full topology preservation on individual structures whilst vastly outperforming the other baselines on overall scene topology. The code is available at: https://github.com/mwyburd/TEDS-Net.

## 1 Introduction

Structural segmentation is a fundamental step in many medical image analysis pipelines, used frequently in diagnostics and for monitoring disease progression (Ganguly et al., 2010). Reducing a medical image into a binary segmentation map by grouping together pixels of the same structures, can help simplify clinically-required measurements necessary for downstream analysis tasks. However, for such measures to be meaningful, the segmentation must be highly accurate and represent the true anatomy of a target structure.

**Fig. 1:**
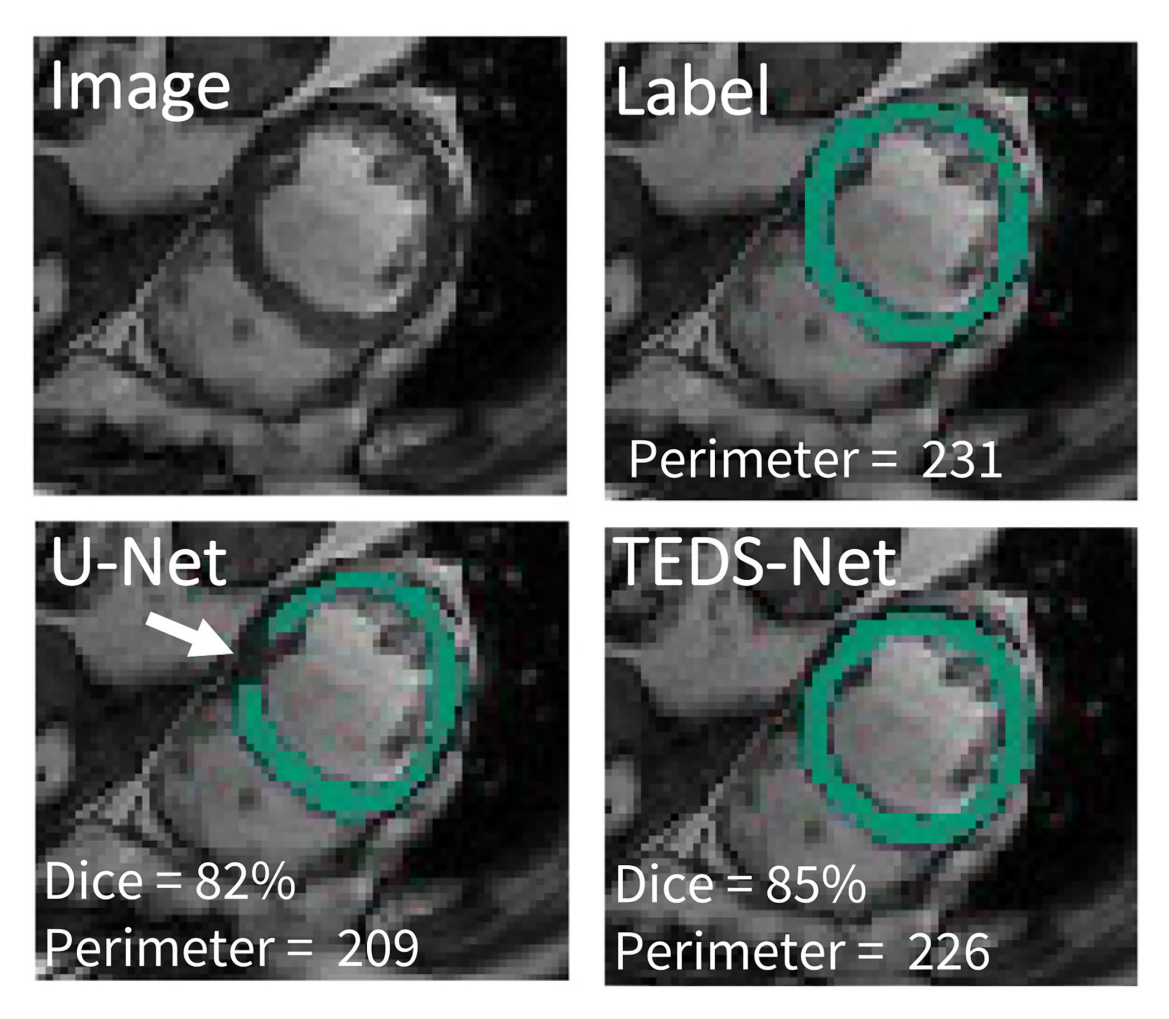
Example of topological errors found when segmenting medical images using traditional CNNs, i.e. a U-Net style architecture, trained with a pixel-wise loss function. Here we show myocardium segmentations from 2D MRI short axis slices. The gap found within the U-Net example (indicated with the white arrow) changes the topology and interferes with automated measures of perimeter.

For a medical segmentation to be considered anatomically plausible its *topology* should be equivalent to that of the true anatomy. The topology, or *higher-level structure*, describes the overall shape properties of a structure that are preserved under continuous deformations, e.g. without joining or tearing, or closing or creating holes within the object. In short, it is a measure of the number of connected components and holes within a structure. For example, the heart’s myocardium label from the 2D cross section shown in Fig. 1, can be described as a single connected component with a two-dimensional hole. In such a 2D cross section, if the myocardium segmentation had multiple components or multiple holes, its topology would not match that of the true anatomy and it would be considered anatomically implausible. In clinical analysis pipelines, myocardium segmentations may be required to calculate measures of circumferential strain or wall thickness, commonly used for the diagnosis of hypertrophic cardiomyopathy (Brady et al., 2023; Corona-Villalobos et al., 2013), which can be highly unreliable to perform if topological errors are present. In this work, we delineate complex structures by continuously deforming simplified representations with the desired topological features, to ensure that the final segmentations have the correct topology.

Over the last decade, the time-consuming task of manual segmentation is rapidly being replaced by automated computation tools including deep Convolutional Neural Networks (CNNs), which are now the basis of most newly developed segmentation algorithms (Clough et al., 2020; Litjens et al., 2017). Traditionally, CNNs perform segmentation tasks by assigning a class probability to each pixel within an image, a process known as *semantic* segmentation. Deep CNNs apply a series of convolutions to an input image, learning both local and global image features so that the prediction for each pixel is based on its surrounding neighbourhood. However, as these networks are typically trained and evaluated using pixel-wise loss functions, such as Dice loss or Binary Cross Entropy (BCE), the higher-level structural information is often neglected, which can lead to anatomically implausible segmentation results.

Fig. 1 shows an example of the type of topological errors that can be produced when using traditional CNNs, e.g. a U-Net style network (Ronneberger et al., 2015), that are solely optimised with pixel-wise loss functions (e.g. Dice loss). These errors can interfere with the clinically-required measures of wall thickness and in automated myocardium perimeter tracing (Samuel et al., 2023) defeating one of the primary purposes of the segmentation. Moreover, it should be noted that the impact of these topological errors is not reflected in the relatively high Dice score, illustrating the limitation of optimising and evaluating with such pixel-wise functions. Therefore, when performing segmentation tasks, there is a need to evaluate the topological performance in addition to pixel-wise accuracy, for a more thorough assessment of the segmentation quality.

In recent work, topology-aware loss functions for segmentation tasks have been proposed to overcome the pixel-wise limitations (Clough et al., 2020; Hu et al., 2019; Shin et al., 2020; Shit et al., 2021; Byrne et al., 2021, 2022), and have been shown to help decrease but not always eliminate, the frequency of topological errors. Therefore, there is a need for more explicit anatomical constraints within deep segmentation networks to maintain topology, ensuring that the segmentations are appropriate for some of the necessary downstream tasks.

To enforce the preservation of topology, topology-preserving deformation fields are commonly utilised, with methods incorporating such fields becoming increasingly popular in both the machine learning registration and segmentation communities (Jaderberg et al., 2015; Wyburd et al., 2021; Dalca et al., 2018). However, in our previous work we showed that the guarantees afforded by continuous topology-preservation begin to breakdown when working with fields in the discrete domain (Wyburd et al., 2021). We proposed TEDS-Net: Topology Enforcing Diffeomorphic Segmentation Network, a segmentation network used to deform a prior shape through learnt topology-preserving fields, which accounts for the discrete limitations through novel modifications, resulting in 100% topology preservation in myocardium segmentations.

This paper extends TEDS-Net, previously presented at the Medical Image Computing and Computer Assisted Interventions (MICCAI) conference 2021 (Wyburd et al., 2021). The methodology has been kept largely the same, but with new theoretical examples and experiments. First, using a simple 1D example, we illustrate how the properties of continuously-defined fields are not guaranteed when generating such fields in the discrete domain. This is supported by hyper-parameter exploration that shows without our additional modifications, our generated fields are no longer topology-preserving. Further, we apply TEDS-Net to new datasets with varying topologies and for multi-structure segmentation, to show its generalisability to new structures and more complicated tasks. Through experiments, we add new baselines and more rigorous evaluation measures, to show the limits of TEDS-Net and the importance of topology in medical segmentations. Moreover, we re-define TEDS-Net to *Topology Enforcing Deformation Segmentation Network*, as due to the discrete representation of these fields they cannot be classed as diffeomorphic (as discussed in Section 3.1) and we instead concentrate on generating topology-preserving fields.

## 2 Related work

### 2.1 Semantic segmentation and topology

In recent years, a plethora of methods have been proposed to encourage semantic segmentation networks to learn topological features, to overcome the limitations of pixel-wise optimisation. For instance, a series of topological loss functions have been designed to work in combination with pixel-wise functions, to encourage networks to focus on the higher-lever shape information (Clough et al., 2019; Shin et al., 2020; Hu et al., 2019; de Dumast et al., 2020; Shit et al., 2021; Byrne et al., 2021, 2022). The majority of these functions have been based on *persistent homology* (PH) and have been shown on a range of computer vision and medical imaging tasks, including cardiac segmentation (Byrne et al., 2021, 2022; Clough et al., 2019). In short, PH measures the topological features of a structure across of range of spatial resolutions, and concludes that persistent features across this range are most likely to represent the true features of a structure (Clough et al., 2020).

In Hu et al. (2019), the authors proposed one of the first methods, referred to as *TopoLoss*, which compares the PH and Betti numbers (further discussed in Section 3.4) between the ground-truth labels and the predicted probability maps. As this method was found to be extremely computationally and time expensive, TopoLoss was only calculated on small patches of the images. This procedure, however, was also found to create small disconnected components throughout images, which have to be removed through post-processing operations, such as morphological opening. Similarly to this work, Byrne et al. (2022) designed a PH-based loss function specifically for multi-class cardiac segmentation in 2D and 3D, which significantly reduced the number of topological errors compared to the U-Net. However, loss functions such as these can only implicitly encourage topology preservation, rather than strictly enforce it.

Higher-level shape information of desired structures has also been incorporated into CNNs using additional networks, tasked with learning the topology (Oktay et al., 2017; Mosinska et al., 2018; Painchaud et al., 2020; Cao et al., 2020). In Oktay et al. (2017) an auto-encoder, trained to extract shape information from a set of anatomical labels, was used to regularise a separate segmentation network. Whereas, in Mosinska et al. (2018) the feature maps from a pre-trained VGG-net were used as descriptors of higher-level shape information and were incorporated into a final segmentation network through a topology-aware loss function. Although these methods report promising results and provide prior knowledge of higher-level structure, it is unclear which shape features the networks focus on, and how relevant they are to the structure’s overall topology. To combat this, Painchaud et al. (2020) incorporated a post-processing topology-correction step, which identifies incorrect configurations based on a set of ‘anatomical criteria’. Any *anatomically-invalid* segmentations are then passed through a VAE pre-trained on topologically correct labels, and the *anatomically-invalid* latent vector is updated to the closest *anatomically-valid* latent representation, updating the segmentation. The authors demonstrated their approach to cardiac segmentation, in both the short and long axes which have varying anatomical criteria.

Although the methods described above provide additional shape information, they are still ultimately limited by the method of labelling individual pixels rather than a whole structure, with of the majority proposed topology-modifications only softly encouraging the networks to focus on higher level structure.

### 2.2 Segmenting through deformations

One of the predecessors to semantic segmentation was deformable models (McInerney and Terzopoulos, 1996). As the name suggests, deformable models warp prior shapes to correspond to an image, using deformation fields learnt through minimising an energy function (McInerney and Terzopoulos, 1996). The theoretical basis behind deformable models has since been integrated into a series of machine learning methods. An example of this is spatial transformer networks (STNs) (Jaderberg et al., 2015). STNs were first proposed to learn affine transformations for classification tasks, but have since been adapted for segmentation (Dinsdale et al., 2019; Zeng et al., 2019; Wickramasinghe et al., 2019; Lee et al., 2019; Zhu et al., 2020). In these methods, a deformation field is learnt through a series of convolutions from an input image, which is then used to warp a prior shape that is similar to the structure of interest. These methods often explicitly incorporate the true topology into the network through the prior shape. However, normally the fields are not encouraged to be topology-preserving and therefore there is no guarantee that the warped structure will be topologically equivalent to the prior.

In atlas-based segmentation, an image is registered to a labelled atlas before back propagating the labels to the image space (Zhu et al., 2020). To perform such tasks, a deformation field describing the transform between an image and the atlas is required. In deep learning-based registration methods, as the registration transform is already known, atlas-based segmentation is often performed as a secondary task or incorporated into the network as an additional loss term to improve registration performance, as done in VoxelMorph’s methods (Balakrishnan et al., 2019). VoxelMorph is a popular brain registration network, which uses a CNN to learn the deformation fields used to register a fixed and moving MRI volume (Balakrishnan et al., 2019; Dalca et al., 2018, 2019).

Recently, VoxelMorph-based methods have been extended to try and guarantee topology preservation by generating *diffeomorphic* deformation fields, pioneering the integration of diffeomorphism into CNNs (Dalca et al., 2018, 2019). Diffeomorphic fields are a type of continuous topology-preserving fields that have become common place in deep learning-based image registration methods (Dalca et al., 2018; Mok and Chung, 2020). Recent work has adapted popular diffeomorphic-registration methods, and proposed joint registration and segmentation of images (Sinclair et al., 2020; Wang et al., 2020; Huang et al., 2021; Zhu et al., 2022), with a focus on topology preservation in segmentations, although the final topology performance is often unreported (Sinclair et al., 2020). However, although in theory, diffeomorphic fields should preserve the topology of a prior, in our previous work we showed that, when working in the discrete domain, the continuous properties of topology-preserving fields can be violated, resulting in incorrect segmentation topology (Wyburd et al., 2021). In a similar style to the methods described above, our method (TEDS-Net) learns a topology-preserving field by applying a series of convolutional layers to an input image, which is then used to warp a prior to perform a segmentation. TEDS-Net utilises the theory behind diffeomorphisms, but with additional novel modifications used to eliminate topology violations in the generated fields that are associated with working in the discrete domain.

To assess whether a field is topology-preserving, the Jacobian determinant at each position should be measured (Karaçali and Davatzikos, 2004). The Jacobian determinants provide an insight into how the field is behaving at a given position: if its value is less than zero, the field folds over itself, thus making it no longer topology-preserving. The additional modifications in TEDS-Net proved pivotal in removing any folding-voxels and hence being topology-preserving (Wyburd et al., 2021). In recent work, topology preservation in an STN has been encouraged through a novel loss term that penalises folding voxels within the predicted deformation field (Zhang and Lui, 2022). However, both the final topology performance and Jacobian determinants are left unreported, and as topology preservation is only implicitly encouraged, it is likely that folding voxels are still present.

## 3 Method

The overall aim of TEDS-Net is to automatically segment an anatomical structure of interest, whilst preserving its known topology. To achieve this, topology-preserving fields are learnt from an input image and used to deform a prior shape that has the desired topological characteristics, to generate a segmentation. In this section, we first discuss the generation of topology-preserving fields in the discrete domain (Section 3.1), before describing how they are integrated into TEDS-Net’s architecture (Section 3.2).

### 3.1 Topology-preserving fields

For a deformation field, **Φ**, to be topology-preserving it must be continuous. That is, it must have a continuous inverse and be bijective (one-to-one mapping) at all locations (Musse et al., 2001; Ashburner, 2007). However, in practice, fields are stored over a discrete grid of points (**x**) where these criteria no longer apply (Karaçali and Davatzikos, 2004). In fact, when describing the properties of a discrete field (**Φ**), we are referring to the nature of the continuous version (**Φ**_*c*_) obtained by interpolating between grid points. Although interpolation generates a continuous field, it does not guarantee a one-to-one mapping or continuous derivatives and, therefore, cannot guarantee topology preservation (Karaçali and Davatzikos, 2004). Therefore, the methods used to generate topology-preserving fields in the continuous domain, may not translate when working and storing in the discrete domain (Karaçali and Davatzikos, 2004). In the discussion that follows, we explore how topology-preserving fields are generated in the continuous domain, i.e. diffeomorphisms, and illustrate how the discrete representation of such fields can change their nature. Further, we introduce the additional modifications used in TEDS-Net, to more strongly encourage topology preservation in discretely represented fields.

An elegant property of topology-preserving fields is that when one topology-preserving field is resampled by another (also known as a composition and denoted °), the resulting field will also be topology-preserving (Ashburner, 2007). This property has commonly been used to generate topology-preserving fields, by first initialising with a field of negligible displacements, that, by definition, is topology-preserving, before resampling it by itself repeatedly, to generate a large-displacement field. This method is known as the Scaling and Squaring approach (Moler and Van Loan, 2003; Arsigny et al., 2006):

(1) $$\begin{array}{l} {\text{Φ}}^{1}=u^{1}(x)+x\\ {\text{Φ}}^{2}={\text{Φ}}^{1}\circ {\text{Φ}}^{1}\\ {\text{Φ}}^{4}={\text{Φ}}^{2}\circ {\text{Φ}}^{2}\\ \;\vdots \\ {\text{Φ}}^{2^{h}}={\text{Φ}}^{2^{h-1}}\circ {\text{Φ}}^{2^{h-1}}. \end{array}$$

Although conventions across the literature vary, throughout this work, we will use **u**^*n*^ to refer to *n*^th^ displacement field and **Φ**^*n*^ for deformation field, where **Φ**^*n*^(**x**) = **u**^*n*^(**x**)+**x** and **x** describes the reference grid where the displacements occur. The initial field used in the Scaling and Squaring method is often described as a velocity field but is actually a scaled copy of a velocity field, representing a displacement that is a discrete approximation to the integration of a velocity field over a small unit of time, *u*(*x*) ≈ *v*(*x*) · *Δt*, as is commonly done in numerical integration. Furthermore, throughout the literature the Scaling and Squaring method is often described as integrating a stationary velocity field over a single unit of time, subdividing this unit interval into 2^*h*^ small time steps. The method described in Eq. (1), is identical to this but we have changed the arbitrary units of time such that each time step is now a single unit of time and the entire time interval is 2^*h*^ units. Therefore, rather than describing the composition in terms of small time steps of 1/2^*h*^, we now simply describe the number of compositions performed.

**Fig. 2:**
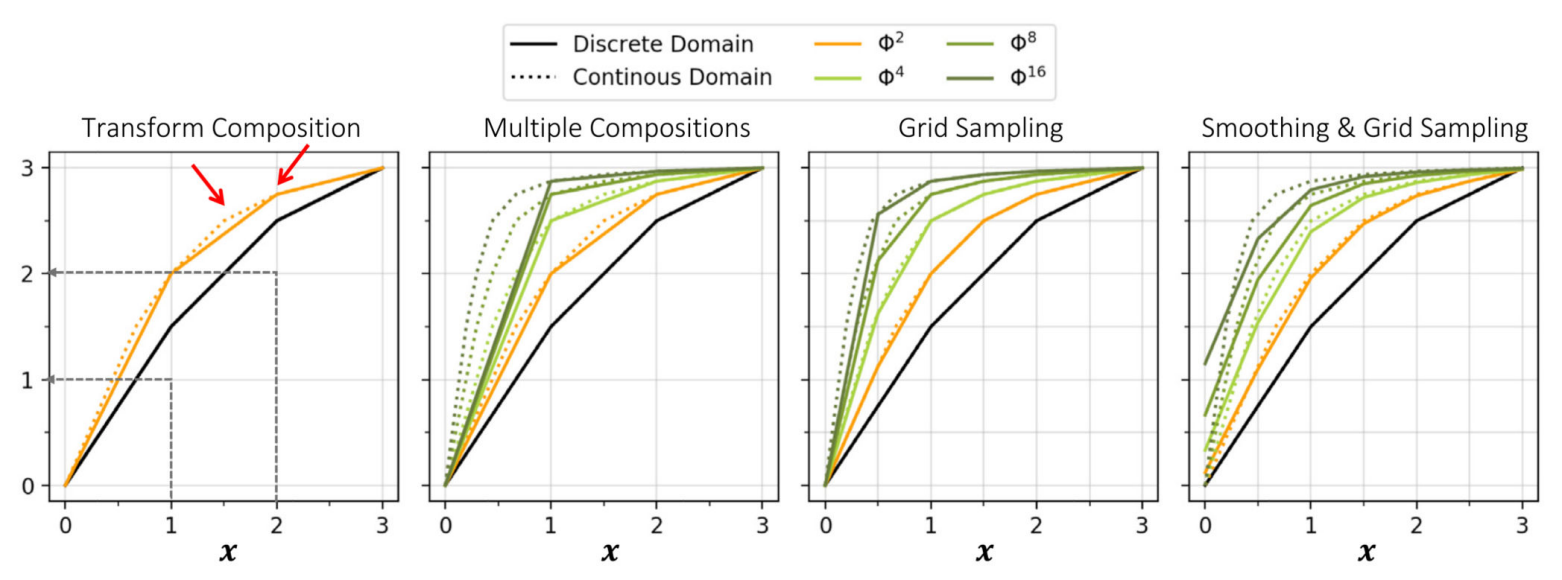
Panels A-D. Panel **A** shows a simple 1D transform **Φ**^1^ and the composition (one application of “scaling and squaring”) obtained through both discrete (solid line) and continuous (dotted line) methods. The grey arrows illustrate the two steps used to calculate **Φ**^2^ in the discrete case at *x* = 1, **Φ**^2^(1) = **Φ**^1^(**Φ**^1^(1)) = **Φ**^1^(1.5) = 2. Panel **B** shows a series of composition layers and Panel **C** and **D** the same transforms after using more finely sampled points, with and without Gaussian smoothing.

Fig. 2 illustrates a simple 1D piece-wise linear function, **Φ**, over grid points: **x** = (0, 1, 2, 3). When performing **Φ**^2^ = **Φ**^1^°**Φ**^1^ = **Φ**^1^(**Φ**^1^) in the discrete domain (e.g. at each grid point) compared to the continuous domain, differences arise, as shown in Fig. 2A. Some differences result in gradients being created or removed within the transform (as indicated with the red arrows), changing the direction of the displacements and therefore likely to disturb the underlying topology. These artefacts often occur when storing the interpolated fields (**Φ**_*c*_) in discrete locations, which risks the loss of important transform information between grid points. Moreover, these artefacts risk becoming more distinct after a series of compositions, where the fields are repeatedly interpolated, followed by discrete sampling and storing, further violating the continuous properties. This effect is shown in Fig. 2B, where the created gradient in the discrete domain is much steeper than that in the continuous. These differences show that, the methods used to generate topology-preserving fields in the continuous domain, such as scaling and squaring, may not directly translate into topology-preserving fields in the discrete domain (Karaçali and Davatzikos, 2004). In this work, to try and mimic the topology-preserving behaviour of the continuous representations, we introduce methods to reduce large gradients created between grid points of discrete fields, so that they more closely resemble their “continuous” counterparts.

Decreasing the space between grid points can help to better describe a transform, decreasing the abnormalities created through storing the interpolated fields, as shown in Fig. 2C, where the spacing has been reduced by a half. The modified discrete-domain fields in Fig. 2C more closely resemble their continuous counterparts, and therefore are more likely to exhibit the same behaviour, compared to the discrete fields in Fig. 2B.

Furthermore, by encouraging the fields to be smooth, steep gradients created through interpolation can be reduced. In Fig. 2D, Gaussian smoothing has been applied between each composition layer to smooth the transform between the discrete grid points, to try and overcome the effects of storing at discrete intervals, as demonstrated in previous work (Cardoso et al., 2015). The inclusion of Gaussian smoothing decreases the steep gradients between the discrete positions, however, it also moves the transformed line further from the continuous positions, as seen at *x* = 0.5 for example. Therefore, although these methods do not enforce behaviour identical to the continuous fields, as the artificial steep gradients have been smoothed, they can encourage continuously-defined properties such as topology-preservation.

In this work, as generating topology-preserving fields is a priority, we include Gaussian smoothing. Throughout this work to generate topology-preserving fields, we utilise methods designed for the continuous domain (e.g. scaling and squaring) but with the addition of Gaussian smoothing and reduced grid spacing, to account for the information lost or artefacts created from the discrete domain.

### 3.2 Network architecture

**Fig. 3:**
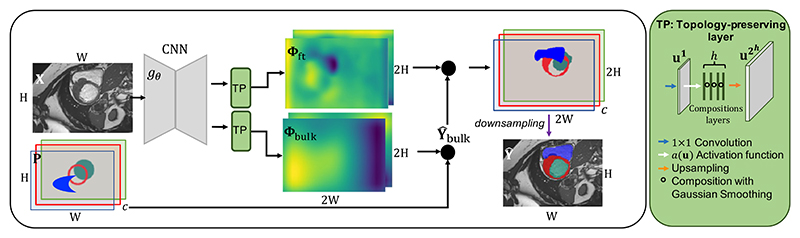
Schematic diagram of TEDS-Net, illustrated for multi-structure segmentation with *c* channels. Two deformation fields are learnt through a series of convolutions applied to an input image, before being encouraged to be topology-preserving through our topology-preserving layers (shown in the green box).

In this section, we introduce the three main components in TEDS-Net, namely: the encoder–decoder; topology-preserving fields layer; and prior sampling. A schematic of the network is shown in Fig. 3. The encoder–decoder network is used to extract relevant features from an input image to predict initial displacement fields. These fields are then encouraged to be topology-preserving before applying them to a prior shape with equivalent topology to the anatomy, generating a segmentation. TEDS-Net learns two deformation fields: one generated at a lower resolution and used to deform the prior into the correct size and location, followed by a second higher-resolution field used to finetune the first deformation. We call these the *bulk* and *fine-tuning* (ft) deformations, respectively.

#### 3.2.1 Encoder–decoder

An encoder–decoder network (*g*), described by parameters *θ*, is used to extract relevant features from an inp t image of dimensions **X** ∈ [*H* ×*W*] to learn two initial fields: $g_{\theta }(X)=\left[u_{\text{bulk}}^{1},u_{\text{ft}}^{1}\right]$. The network resembles that of the U-Net (Ronneberger et al., 2015), as it is made up of a contracting encoder branch that is connected through skip connections to the expanding decoder branches. The encoder branch is made up of a series of convolutional blocks that apply two sets of convolutions with a ReLU activation function and instance normalisation between them (Ulyanov et al., 2016). Initially *f* feature maps are used and between blocks the number of feature maps is doubled and their spatial resolution halved for *l* layers.

Two unconnected decoder streams are attached to the encoder through a bottleneck, which is formed of two convolutional blocks, with skip connections joining each layer of the encoder with corresponding decoder layers. The two streams predict the bulk and fine-tuning fields at different resolutions. The bulk and fine-tuning decoder branches have *l*_bulk_, *l*_ft_ convolutional layers, respectively, where *l*_bulk_ < *l*_ft_. A final 1 × 1 convolution is applied to both branches to reduce the number of features to two to generate a 2D displacement field:$u_{\text{bulk}}^{1}\in {\mathbb{R}}^{2\times (H\times W)/l_{\text{bulk}}},u_{\text{ft}}^{1}\in {\mathbb{R}}^{2\times (H\times W)/l_{\text{ft}}}.$.

#### 3.2.2 Topology preservation

Next, an activation function (*a*) is applied to both initial fields $\left(u_{\text{bulk}}^{1},u_{\text{ft}}^{1}\right)$ to enforce them to be topology-preserving by limiting the maximum displacements to be less than half a grid space, thus preventing grid points crossing over and violating topology. When using a grid spacing of one voxel, this activation function can be written as:

(2) $$a\left(u^{1}(x)\right)=0.5\left(\frac{e^{u^{1}(x)}-e^{-u^{1}(x)}}{e^{u^{1}(x)}+e^{-u^{1}(x)}}\right)=\frac{\tanh\left(u^{1}(x)\right)}{2}.$$

**Fig. 4:**
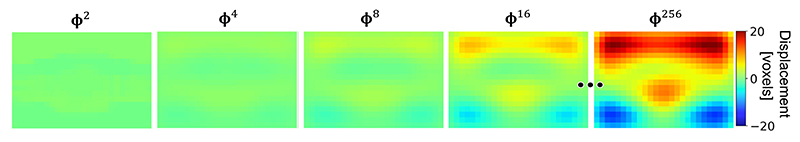
Visualisation of the scaling and squaring approach, shown in one direction. Initially, the fields have negligible displacements, but after composing it by itself *h* = 8 times $\left({\text{Φ}}^{2^{h}}={\text{Φ}}^{256}\right)$ the displacements are amplified.

These fields are then amplified through *h* scaling and squaring layers (adapted from VoxelMorph’s implementation Dalca et al., 2018), which is performed through a series of composition layers described in Eq. (1) and shown in Fig. 4.

Between composition steps, a Gaussian smoothing kernel with a standard deviation of *σ* and size *k*×*k* is applied to reduce the steep gradients created through interpolation and discrete storing, as described in Section 3.1. The impact of *σ* is evaluated in Section 5.2.1. The final composite fields are then upsampled to twice the input resolution, to reduce the grid spacing describing the field and act as an additional smoothing effect: $\Phi_{\text{Bulk}}^{2^{h}}=\Phi_{\text{Bulk}}\in {\mathbb{R}}^{2\times 2H\times 2W},\Phi_{\text{ft}}^{2^{h}}=\Phi_{\text{ft}}\in {\mathbb{R}}^{2\times 2H\times 2W}.$ Different up sampling methods are explored in the Supplementary.

#### 3.2.3 Prior sampling

The bulk displacement field is used to sample a binary prior (**P** ∈ [*H*×*W*]), **Y**_Bulk_ = **Φ**_Bulk_ (**P**), before the resulting image is then resampled for a second time using the fine-tuning field: **Ŷ**= **Φ**_ft_ (**Y**_Bulk_). The prediction (**Ŷ**) is then downsampled to the original input resolution using Max Pooling before a binary threshold of *T* is applied.

### 3.3 Loss functions

To regularise the training of TEDS-Net, both segmentation and field regularisation loss functions are used. Dice loss (ℒ_Dice_) is used to penalise poor overlap between the **Y** and **Ŷ**, in order to improve segmentation performance. A field regularisation loss term (ℒ_Grad_) is used to further encourage smooth deformation fields:

(3) $${ℒ}_{\text{Grad}}=\sum_{i,j=1}^{2H,2W}||\begin{array}{l} \text{Φ}(i+1,j)-\text{Φ}(i,j)\\ \text{Φ}(i,j+1)-\text{Φ}(i,j) \end{array}|{|}^{2}$$

where the spatial gradient is measured at each grid point across the fields, modified from Balakrishnan et al. (2019). As shown in Eq. (3), ℒ_Grad_ is applied to the final fields from TEDS-Net that are twice the resolution of the input: **Φ** ∈ R^2×2*H*×2*W*^. The upsampling smooths the fields and thus the fields more closely resemble their interpolated versions used for sampling, hence reducing the ℒ_Grad_ term.

The network is trained with a weighted combination of these loss functions:

(4) $$ℒ={ℒ}_{\text{Dice}}(Y,\hat{Y})+\alpha {ℒ}_{\text{Grad}}\left({\text{Φ}}_{ft}\right)+\beta {ℒ}_{\text{Grad}}\left({\text{Φ}}_{\text{Bulk}}\right),$$

where the weighting parameters *α* and *β* are used to balance the contribution of the two field regularisation loss terms. The effects of *α* and *β* are investigated in the Supplementary.

### 3.4 Evaluation measures

To evaluate segmentation performance, both the Dice score and Hausdorff distance (HD) are used to compare the ground-truths to the predicted segmentations.

To assess whether a field is topology-preserving, the Jacobian determinant at each position can be measured (Karaçali and Davatzikos, 2004). The Jacobian determinant describes the behaviour around each voxel (x) as being either scaled: |*J*_**Φ**_ (x) | > 1, squeezed: 0 < |*J*_**Φ**_ (x) | < 1, or folded: |*J*_**Φ**_ (x) | < 0. Topology-preserving fields should thus have positive Jacobian determinants at every grid point. Throughout this work we record the percentage of non-positive Jacobian determinants found in each generated field, which is denoted by % |*J*_**Φ**_ | ≤ 0.

To monitor the topology performance, Betti numbers are recorded. Betti numbers are a sequence of numbers that indicate the number of ‘holes’ an object has in each dimension and the total number of connected components. In 2D, a Betti score simply consists of two numbers: the number of components, *b*_0_, and number of holes, *b*_1_. In this work, a hole is defined as any connected component of voxels labelled as background, defined using 4-connectivity, that is surrounded by voxels labelled as foreground. It is important to note that topology does not have a definitive definition in the discrete space as there are multiple definitions of connectivity. Furthermore, the topology of a shape can vary across different image resolutions.

## 4 Experimental set-up

### Overview

We apply TEDS-Net to two open-source datasets to explore different aspects of the model. Firstly, we use a toy-dataset for simple digit segmentation to illustrate that TEDS-Net can segment structures of different topologies within the same training loop. Secondly, we apply TEDS-Net for the task of myocardium segmentation from 2D heart MRI slices in the short axis. In this set of experiments, we evaluate the hyper-parameters that affect the topology-preserving nature of the generated fields and compare TEDS-Net to state-of-that-art (SOTA) baselines. Finally, using the same cardiac dataset, we extend TEDS-Net to perform multi-structure segmentations, where TEDS-Net seeks to preserve topology of the individual structures as well as the combined structure’s topology.

### 4.1 Digit segmentation

#### Dataset

To perform digit segmentation we used the MNIST dataset (LeCun, 1998), which is made up of 70,000 hand-drawn digits from 0 to 9. Traditionally it is used for classification tasks, so to convert it into a segmentation problem the grayscale images were used as the input **X** ∈ [28 × 28] and a binarised version was used for the ground-truth **Y** ∈ [28 × 28], by applying a threshold at 0.5. To increase the difficulty of this task, Fourier-based image augmentation was used to distort the appearance of the digits, by randomly removing lines in the Fourier space representation of the image. The dataset was split into 10,000 for testing and 60,000 for training, where 10% of the training set samples were used for the validation set.

**Fig. 5:**
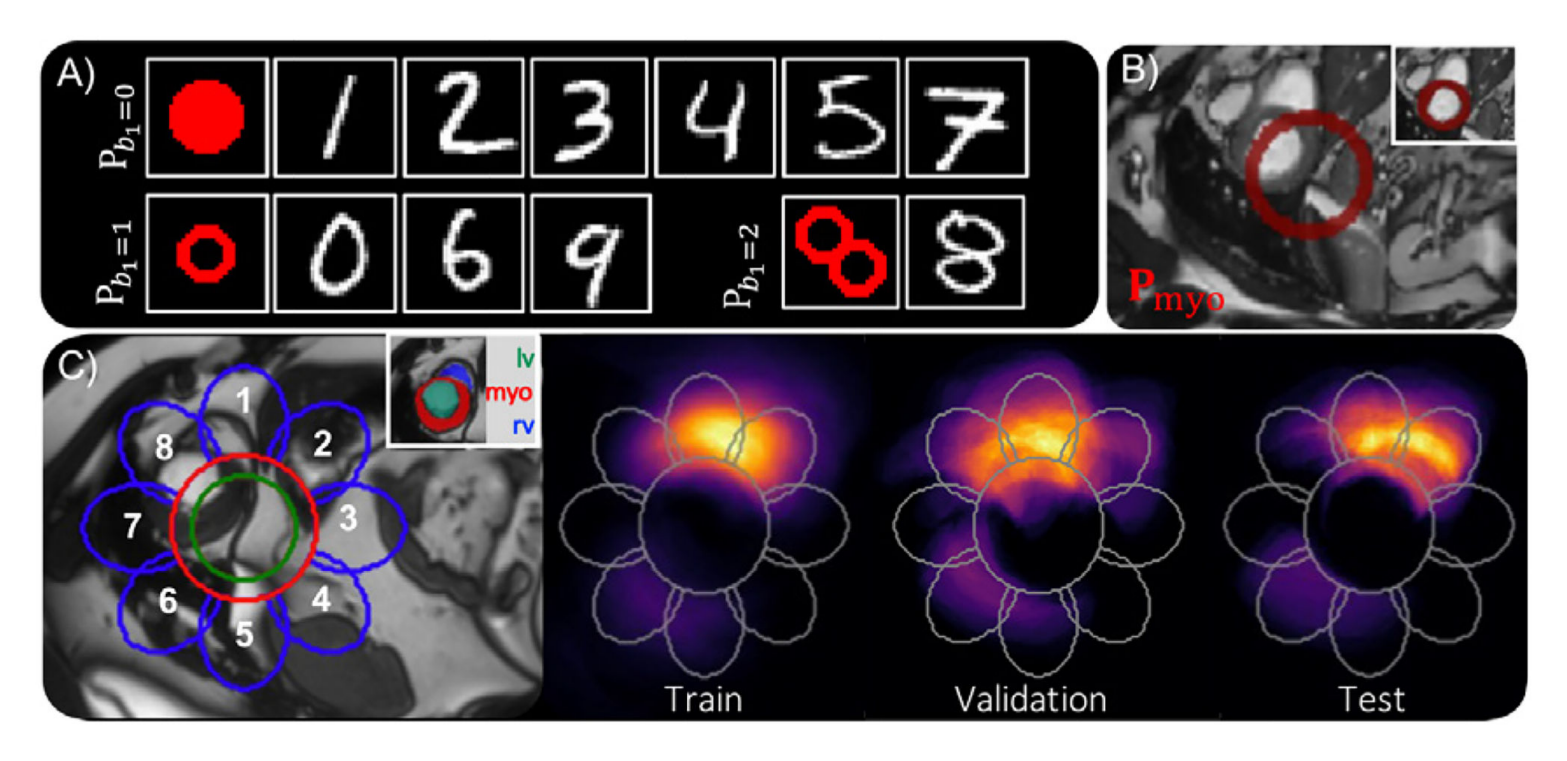
An overview of the priors used for each experiment. Panel **A** shows the priors used for each of the MNIST digits and Panel **B** for myocardium experiments. Panel **C** shows the set of priors used for multi-structure cardiac segmentation and their corresponding label. Further, this figure shows the density of the right ventricle (rv) label in the train, validation and test datasets before augmentation.

The MNIST dataset digits can be classed as three different topologies. The topologies of digits *1,2,3,4,5* and *7*, can be defined by a single component (*b*_0_ = 1) with no holes (*b*_1_ = 0) and therefore have a Betti number: {***B*** = *b*_0_ =1, *b*_1_ = 0}. Whereas, *0, 6* and *9* have a single hole: ***B*** ={*b*_0_ = 1, *b*_1_ = 1} and *8* has two holes: ***B*** = {*b*_0_ = 1, *b*_1_ = 2}. As these topologies are equivalent to a solid circle, a circle with single hole, and a circle with two holes, respectively, these shapes were used as the priors throughout this experiment, as shown in Fig. 5A, and referred to as $P_{b_{1}=0},P_{b_{1}=1},P_{b_{1}=2}$. Each input digit was paired with its corresponding prior at both test and train time, and all digits were trained together.

#### Network implementation

For digit segmentation, the encoder–decoder network was set up with *l* = 2 with *f* = 12 initial feature maps. As the position and size of the digits across the dataset was consistent, the bulk deformation branch was also removed and only a single deformation field was generated from the top layer (*l*_ft_ = 1) of the decoder branch. TEDS-Net was trained for 100 epochs, using the Adam optimiser with a learning rate of 0.0001 and a batch size of 30. The number of compositions was set to *h* = 8, Gaussian smoothing was performed with *σ* = 2 and *k* = 3. As there was only a single field generated, for training we used a loss function: ℒ = ℒ_Dice_(**Y**, **Ŷ**) + *α*ℒ_Grad_(**Φ**_ft_), where *α* = 150.

### 4.2 Myocardium segmentation

#### Dataset

**Fig. 6:**
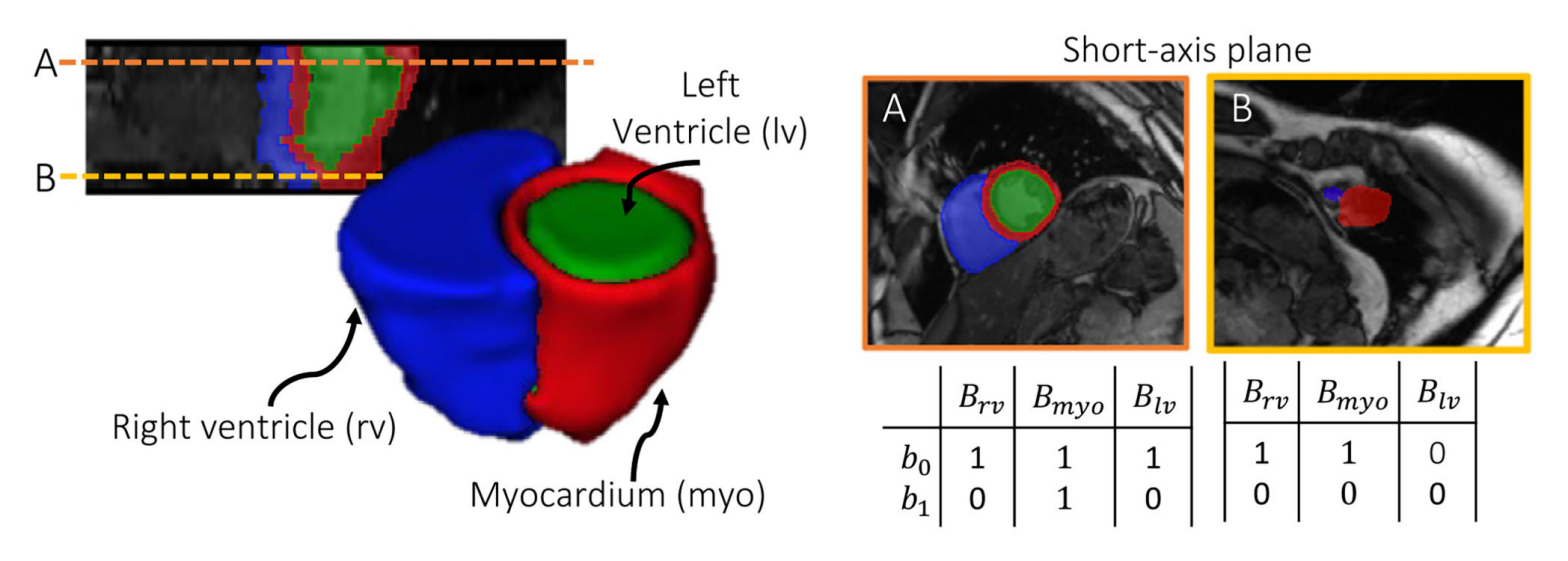
Segmentation of the heart shown in 3D and at two 2D cross sections in the short-axis. Depending on the location of the short-axis slice, the topology of each structure can vary. For TEDS-Net the topology must be known and therefore, only slices with the same topology as shown in Slice A were used across all experiments.

To segment the myocardium, the open-source ACDC dataset was used (Bernard et al., 2018). The ACDC dataset consists of two labelled MRI heart acquisitions per patient, from 100 patients. When viewed in the short axis, the myocardium can be seen to encompass the left ventricle. In the majority of the slices, the myocardium’s topology is therefore equivalent to a hollow circle (*b*_0_ = 1, *b*_1_ = 1), as shown in Fig. 6. From each scan, we selected five short axis slices, where the myocardium had this expected topology. Therefore, for the prior (**P**_myo_), we used a hollow circle with a radius of 35 voxels and a wall thickness of 7 voxels, shown in Fig. 5B and chosen from previous work (Wyburd et al., 2021). Each image was cropped around the centre to 144 by 208 pixels, to reduce the computational demand. Due to the large field of view and consistent scanning protocol used across the ACDC dataset, this cropping was found to keep each myocardium label intact.

#### Image augmentation

Rotations (±5°), translation (±5 voxels), flipping (vertical and horizontal), and resizing (±30%) were applied randomly to increase the dataset, resulting in 8000 images split into 75%, 15% and 10% for training, validation and testing, respectively. Note that the dataset was split into each subset at the subject level, such that images from the same patient did not appear in more than one subset.

#### Network implementation

As the images were unaligned, both the bulk and fine-tuning deformation branches were utilised, to first align and scale the prior, followed by a second deformation to fine-tune the segmentation. The encoder–decoder network was set up with *l* = 4 layers, with the decoders branching off at *l*_bulk_ = 4, *l*_ft_ = 2 and initialised with *f* = 12. Each branch went through *h* = 8 composition layers and the resulting field was upsampled to twice the input resolution and a threshold of *T* = 0.3 was applied. TEDS-Net was trained for 200 epochs, using the Adam optimiser with a learning rate of 0.0001 and a batch size of 5.

#### Hyper-parameter exploration

TEDS-Net is designed to preserve the topology of a prior by generating topology-preserving fields, characterised as having no folding voxels. This is facilitated by generating topology-preserving fields through traditional methods, e.g. scaling and squaring method, and further reinforced through our additional modifications. Across our additional modifications, we identified three parameters that play a crucial role in determining whether folding voxels are present; (i) the *σ* of the Gaussian smoothing kernels used between each composition layer, (ii) the field regularisation weighting parameters (*α* and *β*), and (iii) the interpolation methods used in upsampling, compositions, and image resampling. In our previous work (Wyburd et al., 2021), the removal of either the Gaussian smoothing or the field regularisation terms were found to induce folding voxels within the deformation fields, resulting in violations in topology preservation. We further investigate these parameters here. Across the hyper-parameter explorations we used *h* = 8, *α* = *β* = 10,000, *σ* = 2 and linear interpolation for all sampling, unless otherwise explicitly stated.

Further, in order to utilise the Scaling and Squaring method for the generation of a topology-preserving field, the initial field must also be topology-preserving. To achieve this, we used an activation function (*a*(**u**)(**x**)) as described in Eq. (2). To explore the impact of *a*, we removed it from the architecture and evaluated the performance with and without the additional modifications.

#### Baseline implementation

For the ACDC segmentation, we compared the performance of TEDS-Net with the performance of several baselines, namely: the standard U-Net (Ronneberger et al., 2015); a transformer network Swin-Unet (Cao et al., 2021); VoxelMorph (Dalca et al., 2019); and, using the topological loss function, TopoLoss (Hu et al., 2019).

The U-Net and Swin-Unet were primarily used as a comparison of segmentation performance, trained solely on Dice loss, whereas VoxelMorph, was used to compare the deformation fields with respect to topological preservation and TopoLoss the topology performance. Details of the baseline implementations are provided in the Supplementary.

#### Extension: Detecting the perimeter

Circumferential strain represents the change in myocardium circumference during the cardiac cycle (Johnson et al., 2019). To measure this change, the perimeter of the myocardium is usually manually annotated and tracked across the cardiac cycle (Samuel et al., 2023; Johnson et al., 2019). Automated segmentation removes the need for time-consuming manual annotations. However, for this to succeed the automated method needs to accurately detect the perimeter. To investigate if a segmentation is fit for this purpose, we automatically contoured the predicted myocardiums using a 4-neighbourhood connectivity (Van der Walt et al., 2014) and compared the measured perimeter between each network. From these measurements, we calculated the relative perimeter error for each heart slice; the ideal contour would have an absolute error of 0.

#### Multi-structure cardiac segmentation

##### Dataset

We demonstrated our approach on multi-structure segmentation, using the same ACDC dataset (as described above) but now segmenting the whole heart structure from 2D short-axis slices of the heart, which is made up of the myocardium (myo), right ventricle (rv) and left ventricle (lv). A cross-section of the heart is shown in Fig. 6, displaying how different short-axis slices of the heart can contain the same structures but with different topology. As TEDS-Net requires prior knowledge of each structure’s topology, we only selected slices from our ACDC dataset where the topology of each structure was as follows: ***B***_myo_ = {*b*_0_ = 1, *b*_1_ = 1}, ***B***_rv_ = {*b*_0_ = 1, *b*_1_ = 0} and ***B***_lv_ = {*b*_0_ = 1, *b*_1_ = 0.}

#### Prior shape

When working with multiple anatomical segmentations, it is vital that not only the individual structures have the correct topology but their combined topology, which we will refer to as *scene* topology, is also preserved. As shown in Fig. 6, in the short axis view of the selected slices, the lv fills the entire myo and the rv is always touching the outside of the myocardium ring, generating an overall scene Betti score of ***B***_scene_ = {*b*_0_ = 1, *b*_1_ = 0}. By designing a set of prior shapes with a combined topology of ***B***_scene_ and with the individual channels corresponding to topologies of [***B***_myo_, ***B***_lv_, ***B***_rv_], TEDS-Net should be able to preserve both scene and individual topology.

Across the dataset, the heart slices were unaligned and in different orientations, and therefore the position of the rv relative to the myo varied, as shown in Fig. 5C. Due to this inconsistency, we investigated whether the position of the **P**_*rv*_ relative to the **P**_*myo*_ would affect the segmentation performance. We designed a set of priors where **P**_*lv*_ and **P**_*myo*_ were kept the same but the position of **P**_*rv*_ around **P**_*myo*_, was varied (shown in Fig. 5C). Eight networks were trained, one for each of the right ventricle positions, and the performance of each evaluated on the validation dataset.

#### Network implementation

Moving from single to multi-class segmentation requires very little adaptation of TEDS-Net. The number of channels in the prior and output are increased to *c* = 3, one for each cardiac structure: **P** ∈ ℝ^*c*×*h*×*w*^, **Y** ∈ ℝ^*c*×*h*×*w*^. However, importantly, the number of fields learnt stays the same, as the same field is used to deform each channel, to help preserve the topological relationship between channels. For multi-structure segmentation, the final image downsampling was changed from MaxPool to linear interpolation, as when working with multiple structures that share boundaries, MaxPool often leads to overlapping structures, violating topology. For the task of multi-structure segmentation, TEDS-Net is compared to the same baselines as described in Section 4.2 using the same hyper-parameters.

## 5 Results and discussion

### 5.1 MNIST

**Fig. 7:**
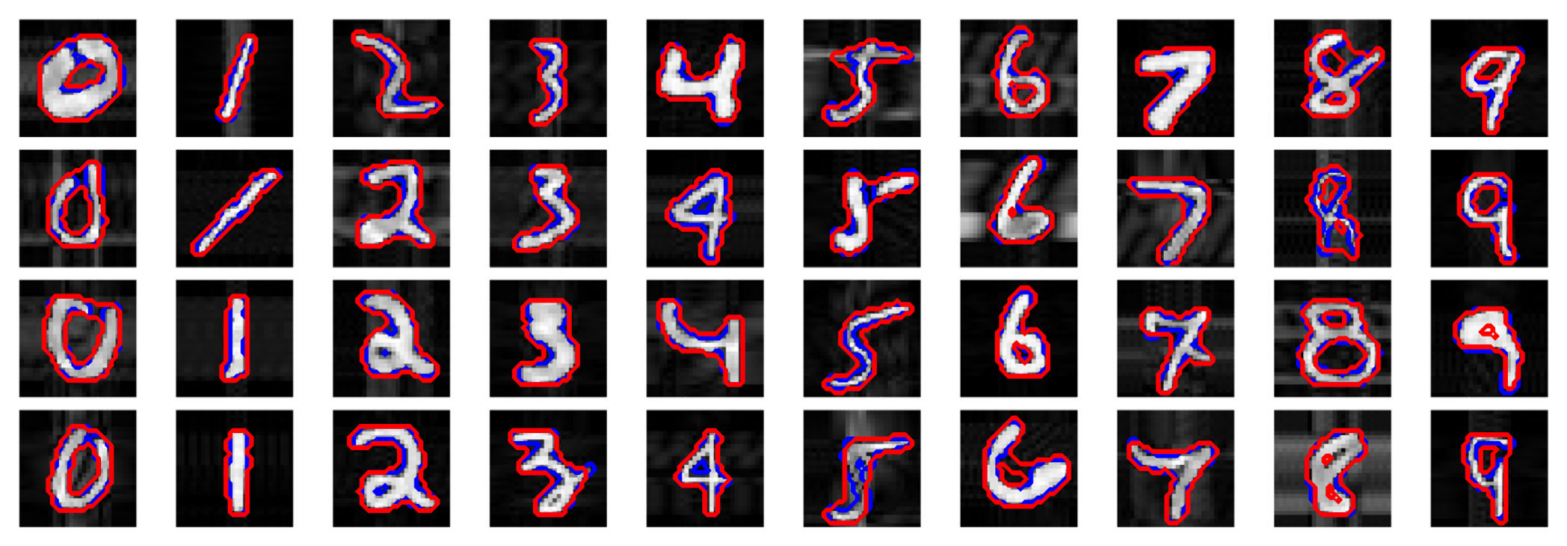
MNIST digit segmentations using TEDS-Net. The blue lines show the labels (**Y**), whilst the red show TEDS-Net predictions (**Ŷ**).

On the MNIST dataset, TEDS-Net achieved an average Dice score of 0.90 ± 0.04 across all digits, with qualitative examples shown in Fig. 7. By comparison, the U-Net achieved 0.96 ± 0.02 and VoxelMorph 0.82 ± 0.06. As this segmentation task is relatively simple, it is unsurprising that the U-Net significantly (*p* < 0.001, as measured with a paired t-test) outperforms TEDS-Net as pixel labelling for such task is much simpler than deforming the prior. Moreover, this experiment was predominantly used to assess the behaviour of TEDS-Net with different priors, rather than to achieve optimal Dice performance. TEDS-Net significantly (*p* < 0.001) outperformed VoxelMorph, with VoxelMorph often found to produce bulky segmentations. It should be noted that VoxelMorph is optimised for 3D registration not 2D segmentation as performed in this task, however, we included this comparison to show how other deformation methods perform with simple priors.

The priors used throughout this experiment were basic shapes with the only prerequisite that they had equivalent topology to their paired digit. For instance, the same prior was used for *1, 2, 3, 4, 5, 7* despite the digits looking drastically different. Despite this, TEDS-Net was able to segment each accurately, illustrating the flexibly of the network and that priors of substantially different shapes and orientations (but the same topology) can be used to segment structures to a high standard. The segmentation performance between digits was significantly different (*p* < 0.01, as measured using a ANOVA un-paired multi-comparison test). Digits that more closely resembled the priors (e.g. “1” and “0”) were found to perform significantly better (measured using an unpaired t-test, corrected with Bonferroni (Bonferroni, 1935)) than the more complex digits (e.g. “5” and “9”), as shown in Fig. B.16.

In total, 99.53% of the 10,000 segmented structures from TEDS-Net retained the same topology as their prior. In comparison, the U-Net achieved 92.14% topology accuracy (between the segmentation and the label). Given the simplicity of this task, it may be surprising that the U-Net’s topology performance is not higher, especially given the high Dice score (0.96 ± 0.02), demonstrating that a high Dice score does not always indicate topology performance. Further, VoxelMorph preserved the topology of the prior in 89.9% of the cases, 10% less than TEDS-Net. However, in TEDS-Net 71.43% of the generated fields (**Φ**) contained folding voxels, with the average fraction of folding voxels 4.6% (equivalent to 36 voxels).

**Fig. 8:**
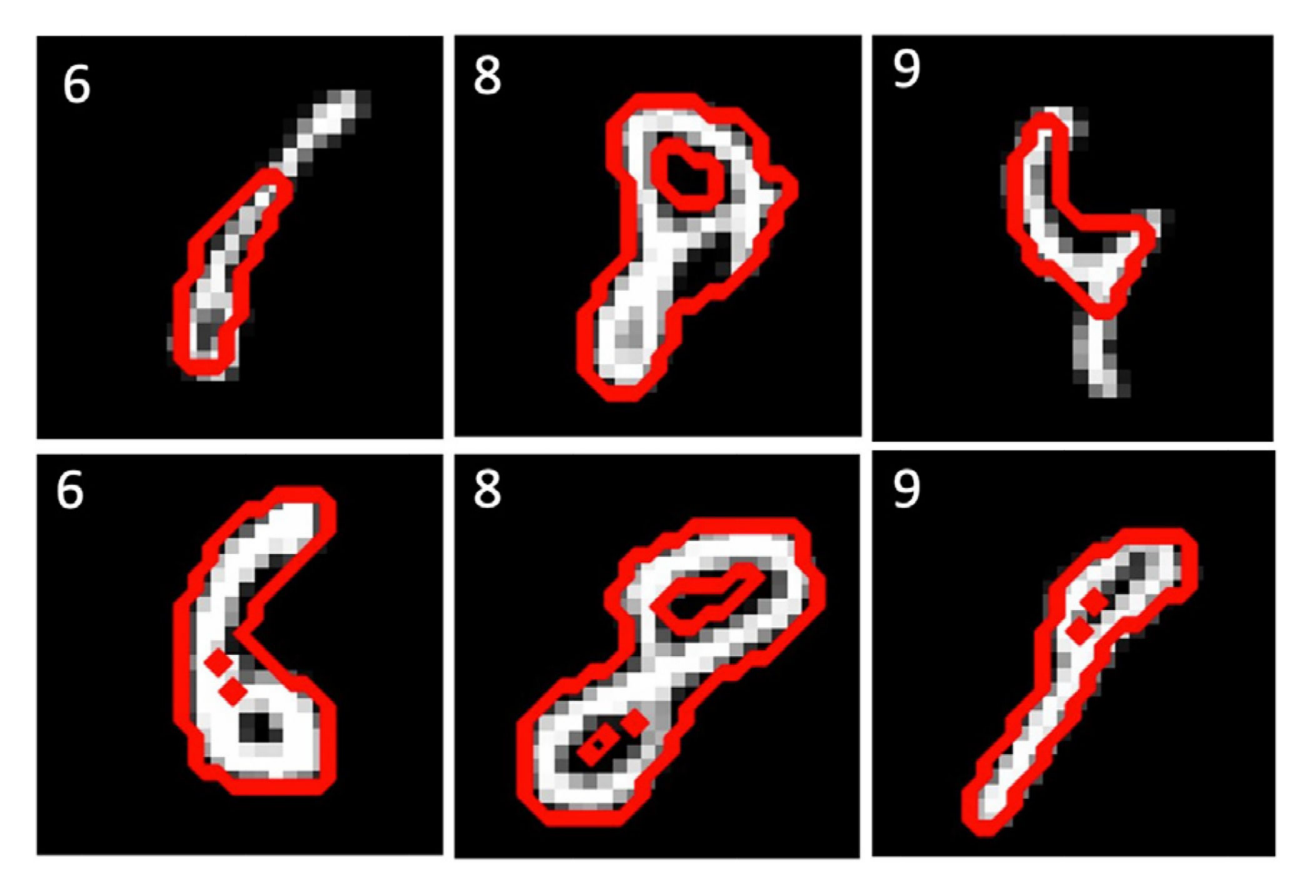
Examples of where TEDS-Net has not preserved the topology of the prior, these examples were randomly chosen.

All the topological errors generated by TEDS-Net were in digits 6, 8, 9, with 33 cases of incorrect topology for 8’s, 11 for 6’s and 3 for 9’s. Fig. 8 shows randomly selected examples of the topological errors from each digit. In the top row TEDS-Net has removed a hole from the prior, whereas in the bottom row, the prior hole has been separated into two holes. These topology violations could be caused by folding voxels, however, other sources, such as the downsampling of the images and thresholding of the final prediction, could also break the topology.

As seen in Fig. 7, the topology of the prior assigned to each digit was not always equivalent to the handwritten digit’s labels due to the natural variation in handwriting, with 24% of the MNIST dataset found to differ from the topology we assigned. Despite this, TEDS-Net predictions were found to preserve the prior topology in the vast majority of the cases (99.53%). Further, Fig. 7 shows that in some cases the Dice performance was negatively impacted to ensure topology preservation, despite the network being partially optimised to minimise Dice Loss, demonstrating how strictly topology preservation is enforced within TEDS-Net. For instance, in the hand-drawn examples of *“0”*, the top of the loop was commonly left open, changing its topology. However, in TEDS-Net’s segmentation’s the loop was completed, following the contours of the shape. This property of TEDS-Net could be desirable in medical segmentations, when shadows or artefacts are obstructing parts of the structure of interest. A similar pattern can be seen for digits *“8”* and *“9”*, and in *“5”*, where the labels often show two disconnected components. Natural variation in handwriting is particularly seen in digits *“2”* and “4”, where the digit can either be drawn as a single component with no hole or a component with a 2D-hole within it. In Appendix B, we investigate swapping the prior shape at inference to the alternative digit topology. The prior shape’s topology is preserved despite the network not seeing this digit-to-prior pairing in training.

This experiment demonstrates TEDS-Net’s ability to segment structures of multiple topologies to a high standard within the same training cycle by using paired priors. Moreover, it also shows how strictly our network enforces topology preservation and that this property often overrides the underlying topology seen in an image. Further, it shows that our method is only suited to segmentation where the topology is always known, for example, organ segmentation where the anatomy should always be consistent, and is clearly advantageous when topology preservation is key.

### 5.2 Myocardium segmentation

#### 5.2.1 Hyper-parameter exploration

##### Gaussian smoothing

**Fig. 9:**
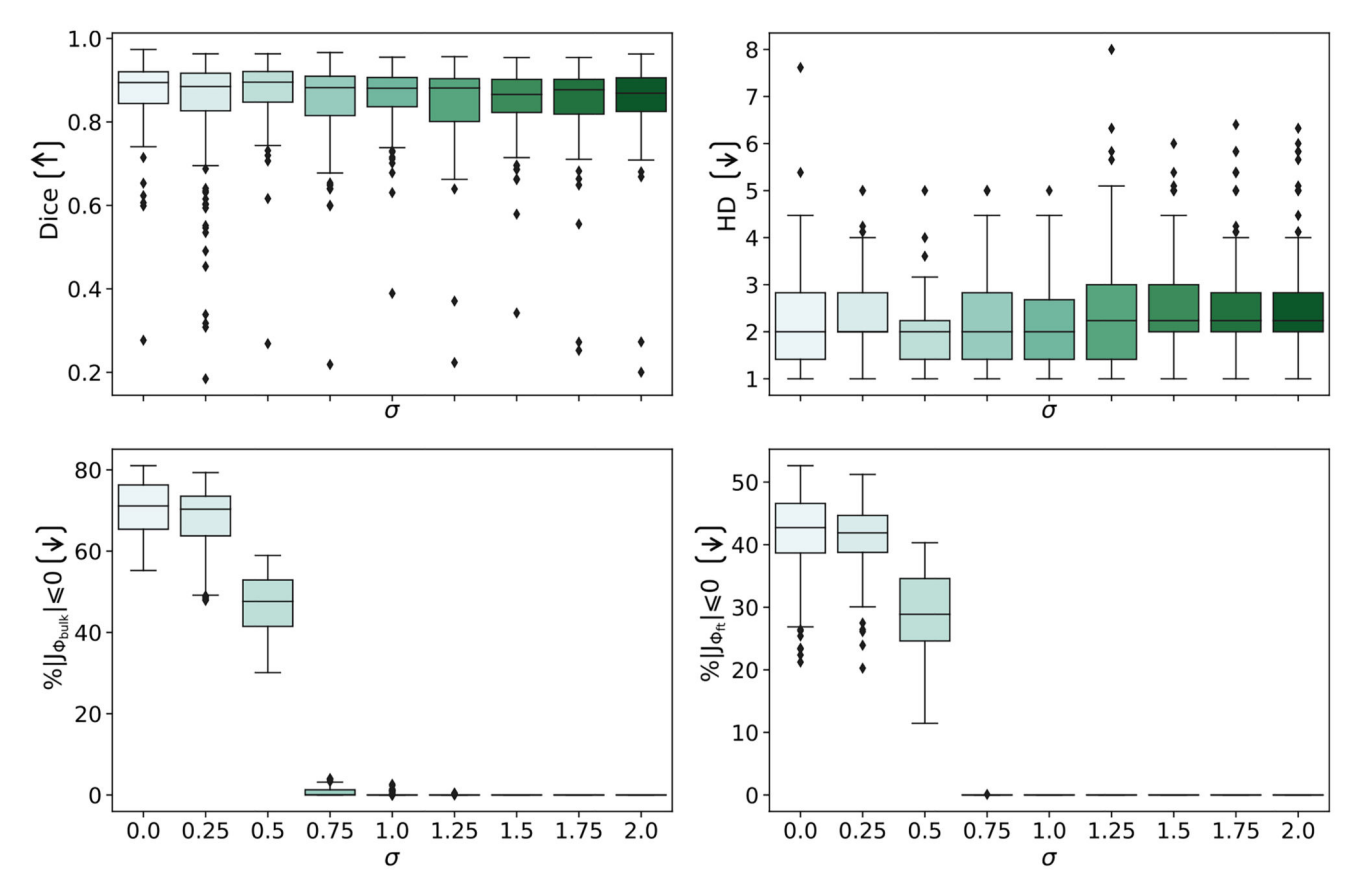
The effect that *σ* within the Gaussian smoothing kernel played on segmentation performance, shown with the average Dice score and Hausdorff distance. The bottom row shows *σ*’s role in preventing folding voxels, where folding voxels are given by % |*J*_**Φ**_| ≤ 0 in the bulk (left) and fine-tuning branch (right).

To smooth the fields generated at each composition layer, a Gaussian smoothing kernel with sigma of *σ* (given in pixels) was applied. Here, we investigate the effect that the value of sigma had on the generated fields and the segmentation performance, as shown in Fig. 9.

The segmentation performance was relatively unaffected across different values of sigma, with no significant differences found in Dice or HD between different values of sigma (measured using ANOVA). This illustrates that increased smoothing of the deformation fields does not negatively impact the final warped segmentation. However, a reduced sigma corresponded to the presence of folding voxels, shown by (% |*J*_**Φ**_| ≤ 0) > 0. When *σ* ≤ 1.25 a high percentage of non-positive Jacobian determinants were found, which often corresponded with topological errors. For instance, when no smoothing was applied (*σ* = 0), folding voxels were present in the fields generated for every input, which resulted in 4.6% of cases having topology errors. The same pattern was found when using *σ* = 0.25, where 11.3% of the segmentations were topologically incorrect.

When *σ* ≤ 1.25, a larger percentage of folding voxels were found in the bulk field compared to the fine-tuning field. The aim of the bulk field is to localise the prior, and therefore, it often contains larger displacements, which can risk large gradients being created between grid points. Therefore, it is likely that the bulk field benefits more from smoothing and thus why more folding voxels are present if the smoothing is removed.

As folding voxels were present when no or low smoothing is applied in the composition layers, this shows that additional smoothing modifications are required when using methods such as the scaling and squaring approach in the discrete domain to more strictly encourage topology-preservation. High segmentation performance, without the presence of folding voxels, was found when using *σ* ∈ [1.25, 2.0]. *Since σ* = 2.0 *performed marginally, but not significantly, better than the other values, it was used for the rest of the experiments*.

##### Interpolation methods

Interpolation of the generated fields risks violating key topology-preserving properties, as discussed in Section 3.1. Across TEDS-Net, there are three instances where interpolation is applied: (i) field composition layers, (ii) super up-sampling, and (iii) when resampling the prior. To explore the effect that the sampling had on topology-preservation, three interpolation methods, namely: linear, bicubic, and nearest neighbour, were used at each of these three instances, as shown in Fig. C.18, Appendix C.1. Setting the interpolation method for both the composition layer and prior resampling to either *linear* or *bicubic* resulted in strong segmentation performance and no folding voxels when used in combination with linear up-sampling. *As linear interpolation resulted in marginally better segmentations, it was used for all interpolations for the rest of the experiments*.

##### Field regularisation

Field regularisation loss functions are commonly used in registration formulations and spatial transformer implementations, to encourage smooth deformations (Dalca et al., 2018; Balakrishnan et al., 2019; McInerney and Terzopoulos, 1996). In Appendix C.2, we investigate how adjusting the contribution of the two field regularisation loss weighting parameters, *α* and *β*, affects the nature of both the fields and the segmentation performance, shown in Fig. C.19. Unsurprisingly, removing the regularisation term from either branch (*α* = 0, *β* = 0), resulted in folding voxels. *The optimal regularisation weights were found to be α* = *β* = *10,000, and thus were used for the rest of this work*.

##### Activation function

Removing the activation function (*a*), whilst keeping the rest of the modification in place, was found to reduce the Dice score to 0.70 ± 0.13. Moreover, it also introduced folding voxels in both fields, with **Φ**_**bulk**_ and **Φ_ft_** found to contain on average 0.2% and 13.2% folded voxels, respectively. This corresponded to no topology errors. Moreover, the removal of *a* resulted in less folding voxels than low Gaussian smoothing, as shown in Fig. 9. Removing *a*, as well as our other modifications, e.g. ℒ_Grad_, Gaussian smoothing and upsampling, resulted in a high percentage of folding voxels: 60.9% and 81.5% for the **Φ**_**bulk**_ and **Φ_ft_**, respectively. Further, in all cases the model deformed the prior outside of the field of view. This implies that the activation function in combination with our additional modifications is essential to constrain the size of the deformations.

Investigating the hyper-parameters within TEDS-Net illustrates why additional field modifications are required in order to remove folding voxels within a discretely represented field. The backbone of TEDS-Net generates topology-preserving fields using methods designed for continuously represented fields (such as the scaling and squaring approach). However, without our additional modifications (e.g. when *α* = 0, *β* = 0 and *σ* = 0) folding voxels were always found. Thus it is clear that when working in discrete space, further action is required to combat the effect of discrete sampling and representation of fields for the topology to be preserved.

##### 5.2.2 Baseline comparison

**Fig. 10:**
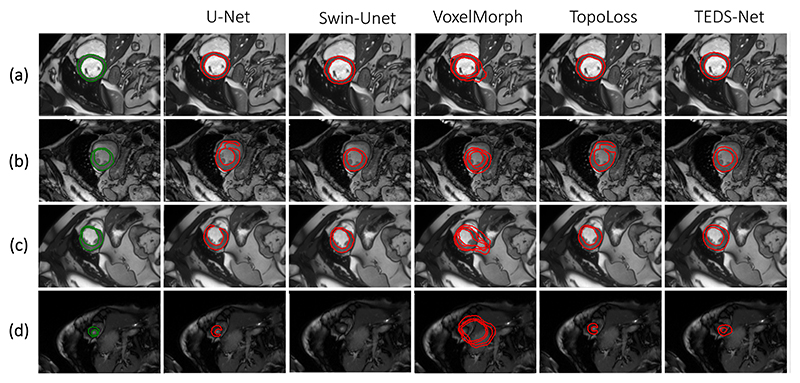
Qualitative performance of myocardium segmentation for TEDS-Net and across the baselines for four different patients (a-d). The ground truth labels (**Y**) and predicted segmentations **Ŷ**) are shown in green and red, respectively.

Table 1 and Fig. 10 display both a quantitative and qualitative comparison of the TEDS-Net myocardium segmentation performance compared to the baselines. The myocardium segmentation performance assessed with both Dice and HD was found to be extremely competitive between U-Net, Swin-Unet, TopoLoss, and TEDS-Net, and none of the five networks were found to significantly outperform the others, as measured with ANOVA.

TopoLoss achieved the joint best segmentation performance, with an average Dice overlap of 89%. As the TopoLoss method used the pre-trained U-Net initially, it is expected that it should perform on par or better than the U-Net. Fine-tuning with TopoLoss did improve the U-Net’s topological performance from 94% to 95%, and was able to correct some of the topological errors found in U-Net (e.g. Fig. 10c). However, in instances where large breaks in the myocardium were present (Figs. 10b and 10d) the topology was not fixed, which may have been particularly challenging to correct using TopoLoss’s patch-based method. Moreover, TopoLoss also required additional post-hoc morphological operations, however, when the same operations were applied to the different methods the number of topological errors increased, illustrating that a unison method is preferred. The myocardium segmentations from Swin-Unet contained the highest number of topology errors. This may be surprising as it is thought that transformer networks learn global structures better than traditional CNNs. TEDS-Net performed worse, in terms of Dice, for cardiac slices at the bottom of the heart, where the myocardium is smallest as shown in Appendix E. This may be due to difficulties in reducing the size of the prior whilst retaining smooth and topology-preserving fields.

VoxelMorph’s implementation was found to produce small topological errors in two of the test images. However, the corresponding predicted fields contained no folding voxels. At closer inspections these detected “holes” within the myocardium would not be classed as a “hole” if defined using 8-connectivity instead of 4-connectivity. TEDS-Net outperformed VoxelMorph in both Dice and HD, which may be a result of the dual branch approach used in TEDS-Net, where the network first learns to localise the myocardium before fine-tuning the segmentation. From inspection of Fig. 10, it appears that VoxelMorph struggles to transform the prior to the correct location and size. More-over, it is important to note that VoxelMorph has been optimised for image registration not segmentation, and this comparison was mainly to address the topology-preserving properties of the predicted fields and the flexibility of the predicted fields.

TEDS-Net was the only method to achieve 100% topology preservation. This achievement coincides with 0% non-positive Jacobian determinants in both the bulk and fine-tuning discrete fields, **Φ**_**bulk**_ and **Φ_ft_**, implying they are topology-preserving.

Although TopoLoss achieved promising segmentation performance, it also required the longest run-times, as shown in Table 1. In comparison, U-Net, VoxelMorph, and TEDS-Net are two orders of magnitude faster, training in 0.22, 0.40 and 0.55 min per epoch, respectively. TEDS-Net’s training time is double that of the U-Net, which is likely to be due to the increased number of parameters in the network due to the two decoder branches and the time taken during the interpolation. Moreover, both TopoLoss and Swin-Unet required a pre-trained network, which was not accounted for in these run-times. The Swin-Unet architecture required 415 × 10^5^ parameters, 60× greater than TEDS-Net, and this drastic difference is paired with a longer time per epoch. TEDS-Net’s segmentation performance is competitive to state-of-the-art baselines whilst outperforming them on topology performance, alongside having a relatively computationally undemanding training.

#### 5.2.3 Extension: Detecting the perimeter

**Fig. 11:**
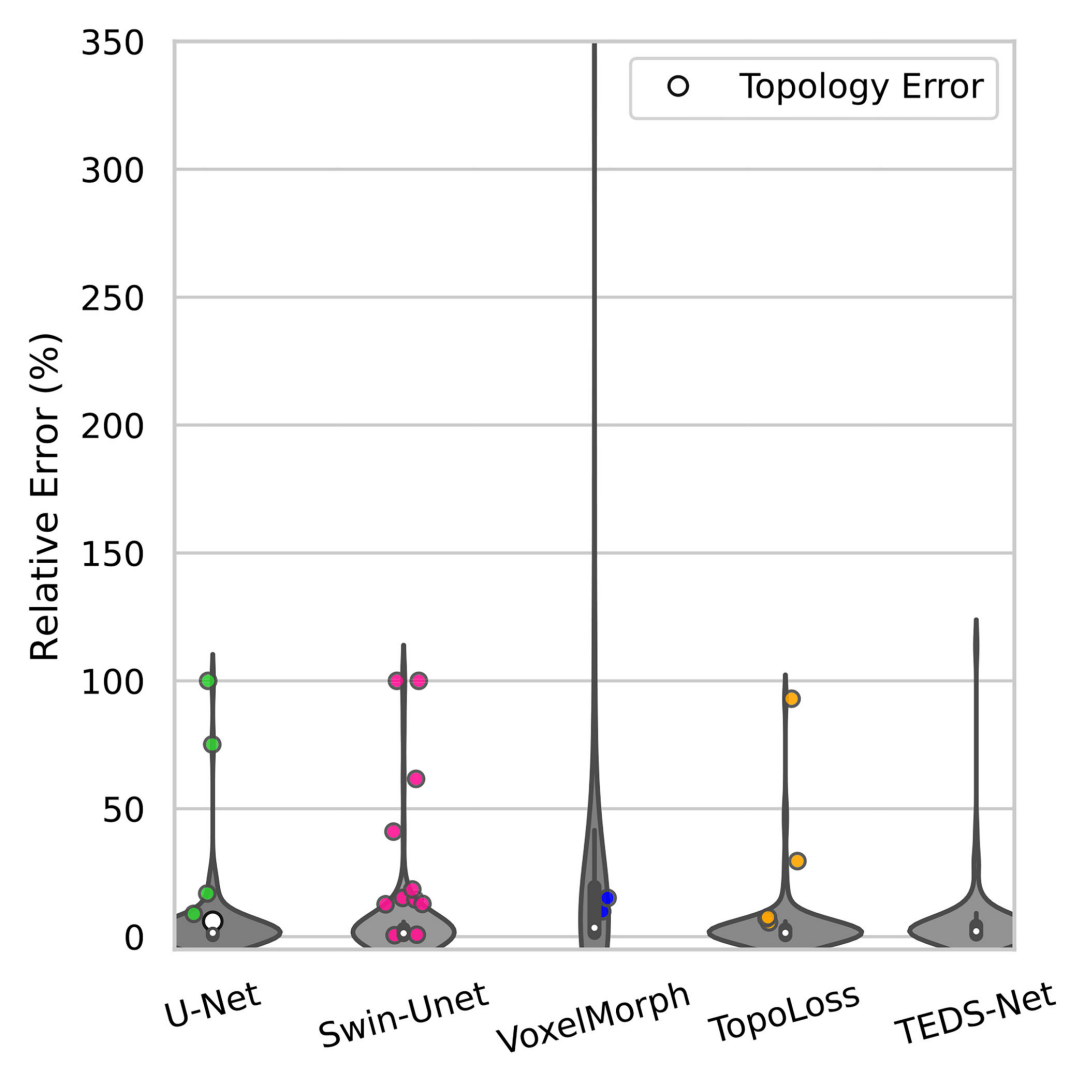
The relative error of automated myocardium perimeter measurements across the five methods. This shows the ratio between the absolute error and the known perimeter value, expressed as a percentage of the known perimeter value. The average myocardium perimeter was found to be approximately 250 voxels. The myocardium perimeter from each prediction was compared to the perimeter of the labels. The mean for each network is shown in white, and predictions with incorrect topology are highlighted.

Fig. 11 shows the difference between the measured myocardium perimeter and its known value across the different networks. For U-Net, TopoLoss, Swin-Unet and TEDS-Net, the majority of the measured perimeters matched closely to the paired labels, shown with absolute errors close to 0, with a small number of outliers for each network. The segmentations with topological errors are indicated and the measured perimeters for these cases are often found to have a large relative error, showing that topological errors can make automated contouring challenging. Despite TEDS-Net’s having achieved the correct topology across all myocardium segmentations, a small fraction of segmentations returned poor perimeter measurements. These cases were from MRI slices near the base of the heart, where the myocardium is smaller and can be more difficult to segment (shown in Fig. 10d) and were consistently poor across the other methods.

Pixel-wise segmentation methods often drop in performance when applied to images in a new domain, e.g. different scanner vendors or clinical cohorts. As TEDS-Net incorporates prior shape information, it may be able to overcome the effects of severe domain shift. In Appendix D, we show that the topology performance of the baseline networks dramatically decreases when applied to a multi-vendor dataset. Whereas, TEDS-Net still achieves the correct topology and outperforms the other networks on Hausdorff distance.

### 5.3 Multi-structure segmentation Prior shape

**Fig. 12:**
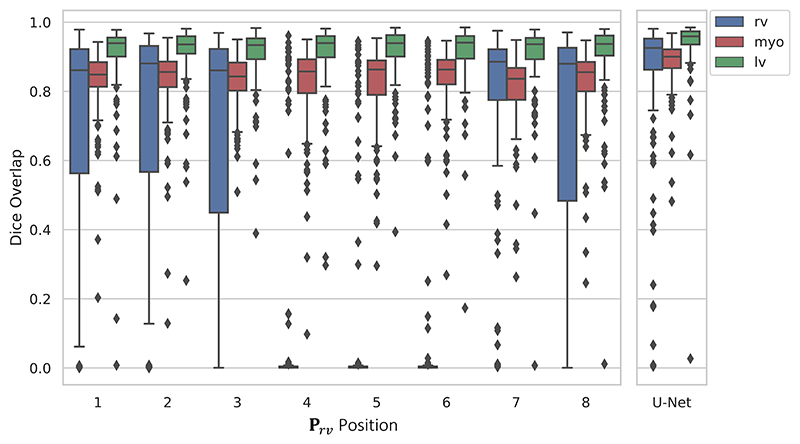
The segmentation performance when **P**_*rv*_ was at each position defined in Fig. 5C, performed on the validation set.

Fig. 12 shows the segmentation performance across the range of rv prior positions defined in Fig. 5C. The majority of the rv labels within the dataset were in positions 1–3 and 8, shown in Fig. 5C. However, when using the **P**_*rv*_ in these positions, the segmentation performance of the right ventricle was found to be highly inconsistent across the dataset. On visual inspection, generally these networks successfully segmented the rv when the labels were found in positions 1 − 3 and 8, but failed in all other cases. This suggests that the network did not learn to rotate the prior, as rotation was not required for the majority of the segmentations. Conversely, only a small fraction of rv labels were found in positions 4–6 and when using **P**_*rv*_ in those positions, the segmentation failed except for a small number of outliers that were found to be in positions 4–6, which also suggests that these networks did not learn to rotate the prior. This might be due to the flipping augmentation, which provides the networks with more rv examples in positions 4–6 as the greatest density of rv labels are found in the opposite positions: 1, 2, 8.

Finally, position 7 contained the lowest density of rv labels, however, the rv segmentation performance was significantly higher and more consistent when using **P**_*rv*_ in position 7, than the other priors. This is likely due to the fact that the network was forced to learn to rotate the prior, as there was minimal overlap between the labels and **P**_*rv*_. Moreover, unlike in the examples above, image flipping should not negatively impact the network’s ability to rotate, as position 7 and its opposite position 3 both have a low density of rv labels. *As position 7 resulted in the best performance, it was used for the rest of the experiments*.

#### Comparison to baseline

Table 2 shows the quantitative results from our multi-structure segmentation on the held-out test set between the U-Net, Swin-Unet, VoxelMorph, TopoLoss and TEDS-Net. The U-Net significantly (*p* < 0.05) outperforms all other baseline networks apart from the Swin-Unet, on rv and myo Dice performance, as measured using FSL’s PALM function for classical multivariate tests, corrected for multiple subjects, multiple network and multiple metrics (Winkler et al., 2016). Interesting, contradicting our previous findings, TopoLoss performs worse on both Dice and Topology than the U-Net, despite being fine-tuned on the U-Net weights. The U-Net and Swin-Unet achieved the highest *scene* Dice, however, it is not significant compared to TEDS-Net (*p* > 0.05). Between the networks, the largest difference in Dice performance is found for right ventricle segmentation. TEDS-Net achieved an average Dice score for the right ventricle of 0.78 ± 0.28, notably with a large standard deviation suggesting that performance is being skewed by outliers, as shown previously in Fig. 12. The median Dice performance for this subset was found to be 0.90 (with an interquartile range of 0.17), a large increase from the mean, further suggesting this measure was being affected by outliers. Through visual inspection, the examples with the lowest right ventricle Dice were found in scans where the right ventricle was prominently in position 3, which would require a 180° rotation of the prior. Therefore, TEDS-Net performance may be limited if the arrangement of the structures is highly varied.

TEDS-Net outperforms the other networks on topology, achieving 100% topology preservation for all three individual structures, which corresponds to the fields generated in TEDS-Net containing no folding voxels, calculated by measuring the Jacobian determinant at all point in the fields. A small fraction (< 1%) of folding voxels were found in 15 of the VoxelMorph predicted fields. VoxelMorph achieved 100%, 73% and 99% correct topology for the right ventricle, myocardium and left ventricles segmentation, respectively. Some of the VoxelMorph segmentations with incorrect topology were generated using fields that contained no foldingvoxels. Further, on visual inspection it was found that these segmentations were classed as a topology mistake due to the discrete definition of connectivity used, as previously discussed in Section 5.2.2.

Across the different models performance, there is a stark difference in the overall *scene* topology with TEDS-Net achieving 70% topological accuracy whereas the next best performing network only achieving 1%. In the U-Net, Swin-Unet and TopoLoss, the most common cause of such errors are gaps found between the tissue interfaces. Whereas, in both the TEDS-Net and VoxelMorph, the *scene* topological-mistakes were found to be overlapping channels, likely created in the downsampling and thresholding. Overlapping is prevented in the other networks due to the Softmax activation function. Moreover, *scene* topology can be used in multiple-structure segmentation, to provide an assessment of the relative position of each structure. As the vast majority of healthy anatomical structures in the body have a known arrangement, the prior knowledge of the anatomy incorporated into TEDS-Net has the potential to guide the arrangement of the structures as well as their individual topology.

## Conclusion

In this work we present TEDS-Net, a segmentation network that delineates structures of interest by deforming a prior shape using learnt topology-preserving fields. This results in anatomically plausible segmentations, which are crucial in many downstream medical imaging pipelines. TEDS-Net achieved 100% topology preservation across the single-class medical imaging tasks, whilst the Dice and HD remained competitive to baseline performance.

Preserving topology through deformation fields is a popular research area in both registration and segmentation tasks. However, here we show that when generating and implementing continuously defined topology-preserving fields in the discrete domain, violations can occur due to storing and applying fields at discrete grid points. To overcome this limitation in TEDS-Net, we introduced additional field smoothing techniques (e.g. field regularisation loss term and Gaussian smoothing between integration layers) and decreased the discrete grid spacing (through increasing the field resolution), to help eliminate folding voxels within the generated fields. Through hyper-parameter exploration, we showed that these modifications are required to generate true topology-preserving fields. It is important to note that our implementation does not guarantee topology preservation but instead strongly encourages it, and this work has shown that the choice of hyper-parameters is important in achieving this goal. Therefore, it is necessary to tune these hyper-parameters for each application and/or dataset.

Throughout this work, to assess segmentation performance Dice, Hausdorff Distance, and topology have been measured. Topology is seldom assessed in deep learning segmentation evaluation, despite its ability to reveal fundamental mistakes within the segmentation. Further, we have shown that topological mistakes are often not reflected in relatively high Dice scores, the most widely used segmentation metric. One example of this is shown in Fig. D.20, where TEDS-Net is outperformed on Dice by two baseline methods that contain large topological errors. Thus, we believe topology should be routinely included in evaluation to more accurately assess segmentation quality. Throughout our experiments, TEDS-Net has outperformed the other baselines on topology performance, however, it has been consistently outperformed by the U-Net (albeit not always significantly) on Dice. The motivation for having topologically correct segmentation is to facilitate further analysis in the medical pipeline, which may be challenging to extract if the topology is incorrect, for example, measuring thickness or surface area, or for automated contouring. In such cases, correct topology is more beneficial than a small increase in Dice, and thus for that task, TEDS-Net would be the appropriate choice of network. However, anatomically plausible segmentations are not always required for downstream analysis. For example, if segmentation is being performed to compute a measure of volume, for instance, to compute ejection fraction in cardiac imaging, a higher Dice score would be preferred over correct topology. Thus, care should be taken when choosing a network to ensure that it matches the downstream requirements, and if topologically correct segmentations are required then TEDS-Net is a suitable choice.

**Fig. 13:**
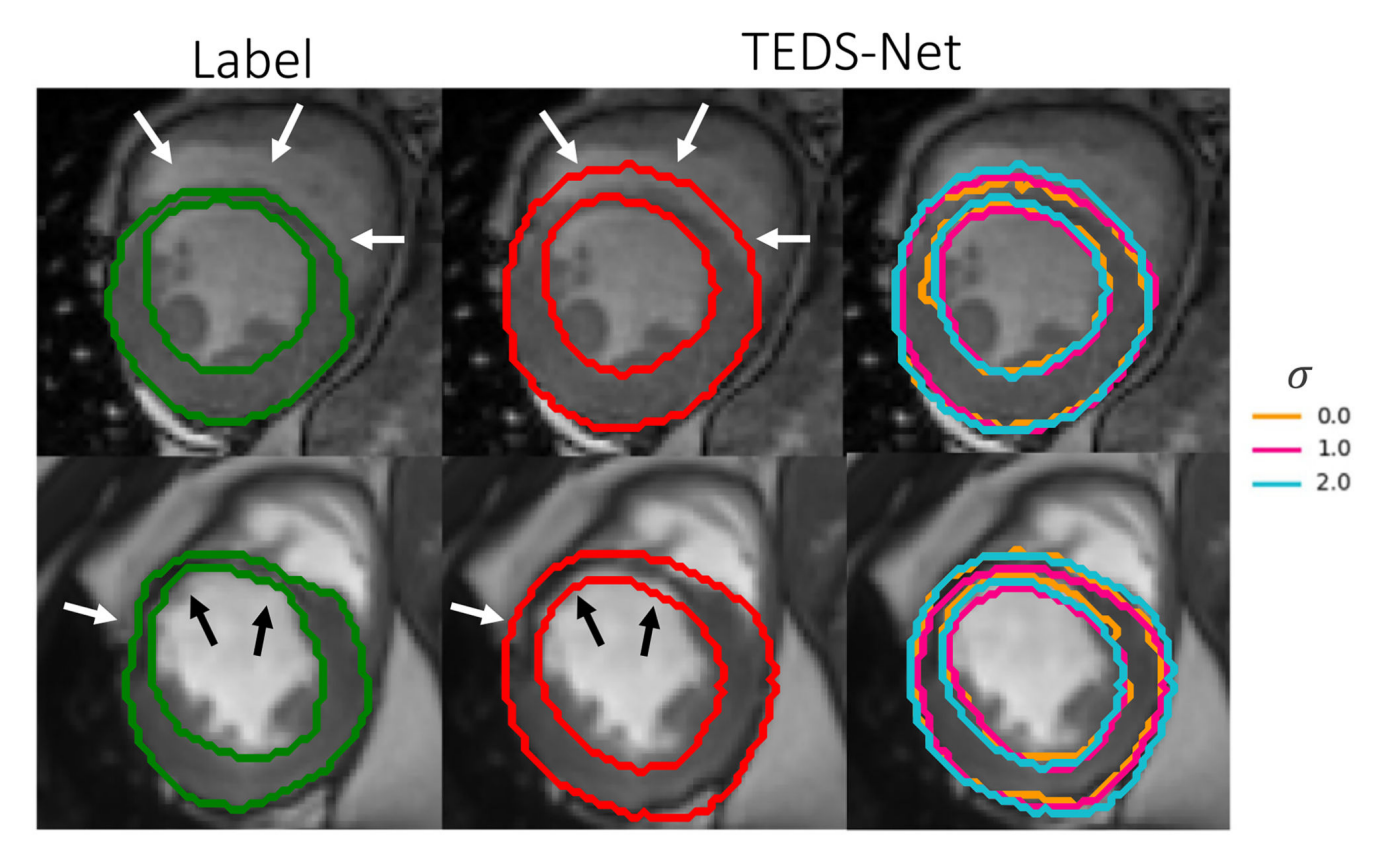
Qualitative examples to show TEDS-Net over-segmenting in regions of thin boundaries. The ground truth is shown in green, TEDS-Net predictions in red and examples at different *σ*’s (*σ* = 0 is no smoothing). Arrows have been added in the same position to highlight the segmentations mistakes at the thinnest regions of the label.

In TEDS-Net’s predictions, the segmentations are often found to be slightly thicker than the labels in thin regions, which can dramatically affect the Dice. This effect is likely due to the additional smoothing modifications introduced to encourage topology-preserving fields. Smoothing within the fields can limit the detail within the deformations, which can result in bulky segmentations. An example of this is shown in Fig. 13, where TEDS-Net over segments the myocardium in thin regions, and therefore, does not accurately represent the true thickness of the structure. To investigate the effect of smoothing and segmentation thickness, Fig. 13 shows TEDS-Net’s segmentations generated from models trained using different *σ*, one of the parameters found to be crucial in removing folding voxels. At the thinnest regions of the myocardium (indicated by the arrows), the predictions generated using no smoothing (*σ* = 0) appear to be marginally thinner than that at *σ* = 2, although still not following the contours of the myocardium label. Therefore, future work should concentrate on improving TEDS-Net segmentation performance. One possible solution for this could be to blur the prior shape, reducing the sharp deformation gradients required at the boundaries (Chakravarty et al., 2013). Reducing the deformation gradient may lessen the requirement for the smoothing modification, which in turn may increase the segmentation performance.

For the task of multi-structure segmentation, we showed that careful consideration of the prior shape is required when the relative positioning of structures is inconsistent across the dataset. In this example, the position of the right ventricle relative to the myocardium was not consistent across the dataset, and we found that different prior locations significantly changes the Dice scores. Ideally, TEDS-Net’s performance should be invariant to the prior shape and size, with the only requirement being that the prior has the correct topology. As TEDS-Net’s performance dropped when the network was required to learn a rotation, an extension to this project could be to initialise with a small network that learns which of the eight prior shapes would perform best for each input, removing the requirement to learn challenging rotations. This adaptation has the potential to increase the overall segmentation performance. Moreover, such addition could also remove the requirement for slice section, as in described in Section 4.2 and performed in both myocardium and multi-structure segmentation. For the case of myocardium, we only trained and evaluated TEDS-Net on slices where the myocardium had the topology of *b*_0_ = 1, *b*_1_ = 1, however, as shown in Fig. 6 in 2D the myocardium’s topology changes at the base of the heart to *b*_0_ = 1, *b*_1_ = 0. Therefore, the ability to automatically choose the correct prior from a set of possible priors could increase the performance and make TEDS-Net more suitable for real world medical imaging tasks. This could further be applied to the MNIST dataset where the digit topology is found to vary due to the natural variation in handwriting.

In this work we have illustrated the ability of TEDS-Net to segment structures with different topologies in two new scenarios, namely: (1) multi-structure segmentations, where structures with different topologies are segmented using multi-channel priors; and (2) digit segmentations where priors are paired to the inputs. These extensions required little modification to TEDS-Net architecture, highlighting TEDS-Net’s flexibility to segment different topologies within the same training cycle. However, a potential limitation of TEDS-Net is that it always requires prior knowledge of the topology of the structure. If the underlying image has different topology to that of the prior, the network prioritises topology-preservation over segmentation performance, which may not be desirable for some applications, e.g. tumour segmentation, where the number of components is unknown.

## Supplementary Material

Supplementary material related to this article can be found online at https://doi.org/10.1016/j.media.2024.103222.

Appendix A-D

## Figures and Tables

**Table 1:** Summary of each network’s performance at segmenting the myocardium from the ACDC dataset. The best result (but not significant) is highlighted in bold. Hausdorff distance (HD) is measured in mm. The training time per epoch and number of parameters used for each method is also reported.

	Dice ↑	HD ↓	Correct topology	Time per epoch [min]	# Parameters [×10^5^]
U-Net	**0.89** ± **0.10**	5.09 ± 3.83	94%	0.22	5.2
Swin-Unet	0.86 ± 0.17	4.37 ± 3.24	89%	1.53	413
VoxelMorph	0.80 ± 0.17	8.81 ± 10.14	98%	0.40	7.3
TopoLoss	**0.89** ± **0.08**	**4.32** ± **5.04**	95%	18.35	5.2
TEDS-Net	0.85 ± 0.14	4.82 ± 3.45	**100%**	0.55	7.1

**Table 2:** Segmentation performance and topology preservation rates between TEDS-Net and the baselines, on each structure: the right ventricle (rv), the myocardium (myo) and left ventricle (lv) as well as the overall scene. The best performance for each measure is highlighted in bold.

Method	Dice ↑			Correct Topology ↑			
	rv	myo	lv	scene	rv	myo	lv	scene
U-Net	**0.88** ± **0.17**	**0.89** ± **0.10**	**0.95** ± **0.11**	**0.96** ± **0.10**	99%	97%	99%	1%
Swin-Unet	0.86±0.19	0.88 ± 0.10	0.94±0.11	**0.96** ± **0.06**	65%	88%	96%	0%
VoxelMorph	0.61±0.33	0.78 ± 0.16	0.90±0.14	0.88 ± 0.11	**100%**	73%	99%	0%
TopoLoss	0.83 ± 0.23	0.87 ± 0.12	0.93 ± 0.13	0.93 ± 0.12	89%	95%	98%	1%
TEDS-Net	0.78 ± 0.27	0.83 ± 0.10	0.93 ± 0.10	0.94 ± 0.05	**100%**	**100%**	**100%**	**70%**

## Data Availability

All data used is open-source and available for download.
