# Supplementary material for: Anatomically plausible segmentations: Explicitly preserving topology through prior deformations
Source: Med Image Anal. Author manuscript; Available in PMC 2026 Mar 14. (PMC7618872; doi:10.1016/j.media.2024.103222)
Supplement: Appendix A-D [file EMS212882-supplement-Appendix_A_D.pdf]

Appendix A. Baseline Training

VoxelMorph. VoxelMorph was used as a baseline to compare both the segmentations and the deformation fields with respect to topological preservation. We used the diffeomorphic VoxelMorph architecture as described in [8]. Unlike TEDS-Net, VoxelMorph is optimised for registration, where 2 volumes (a 'fixed' and 'moving' volume) are passed into the CNN. The network is then tasked with learning the deformation required to warp the 'moving' volume into the 'fixed' volume's space. This method has commonly been adapted to include segmentation, through atlas-based methods, where a labelled atlas is used as the 'moving' volume. However, for this task as an atlas is not available, we instead use the prior ($\mathbf{P}_{\text{myo}}$) as the 'moving' volume, and pass both the MRI slice ($\mathbf{X}$) and $\mathbf{P}$ into the CNN and learn the deformation required to warp $\mathbf{P}$ into the myocardium in $\mathbf{X}$. This is optimised by comparing the warped $\mathbf{P}$ to the known myocardium label $\hat{\mathbf{Y}}$. A combination of Dice loss and field regularisation was used for training $\mathcal{L}_{\text{vm}} = \mathcal{L}_{\text{Dice}} + \omega\mathcal{L}_{\text{Grad}}$.

In previous work, the weighting, ω, between these two loss functions was found to play a key role in balancing registration and field smoothness performance [1, 9]. To allow a fair comparison, we first investigated the effect of this value, as shown in Fig A.1, in order to find the optimal performance for this segmentation task. A trade-off between segmentation performance and field smoothness is shown across values of the regularisation term, with optimal topology performance found at $\omega = 2$, which we therefore continued to use throughout this work. The field weighing terms used for VoxelMorph's implementation are 100 times smaller than that used for TEDS-Net, shown in Fig C.6. This is because the two methods use different grid coordinate systems ($\mathbf{x}$), which results in different orders of magnitudes for the deformations. It should also be noted that VoxelMorph is primarily an image registration method so has not been optimised for segmentation.

U-Net. Similar to the TEDS-Net implementation, both the U-Net and VoxelMorph were trained for 200 epochs, with a learning rate of 0.0001 using the Adam optimiser and a batch size of 5. Both architectures had a depth of $l = 4$ and $f = 12$ initial feature maps.

Swin-Unet. As a second segmentation baseline, we implemented Swin-Unet: a transformer-based segmentation network, which has previously shown state-of-art performance on 2D cardiac segmentation [5]. Transformer networks are often thought to better represent the global information within a task, compared to CNNs, due to the small receptive field within convolutions. Therefore, myocardium topology may be better learnt using Swin-Unet than traditional CNN methods, such as the U-Net. Following the implementation described in [5], Swin-Unet was initialised with ImageNet before being applied to the ACDC dataset. The architecture of Swin-Unet was kept the same as reported in [5] and the ACDC images were padded to 244 × 244 to keep the patch size and number of layers consistent with the original implementation. Swin-Unet was trained for 200 epochs with a batch size of 24, the learning rate was initially set to 0.005, decreasing by 50% every 10 epochs.

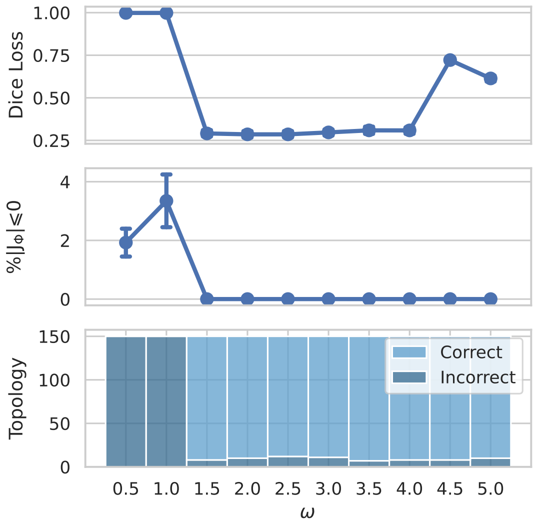

Figure A.1: The effect of the field regularisation weighting in VoxelMorph's implementation. The segmentation overlap (Dice), folding nature of the generating field and topology performance are measured.

TopoLoss. As our final baseline comparison, we used a topology-preserving loss function: TopoLoss ($\mathcal{L}_{\text{TopoLoss}}$) [10]. This compares the topology of a predicted segmentation to the ground-truth segmentation using the concept of persistent homology [10]. As this method is extremely time consuming, it is recommended that it be used on patches of an image and applied to a pre-trained segmentation model. To implement this method, we therefore used the trained U-Net (as described above) and fine-tuned all layers of the network using a combination of $\mathcal{L}_{\text{Dice}}$ and $\mathcal{L}_{\text{TopoLoss}}$: $\mathcal{L} = \mathcal{L}_{\text{Dice}} + \epsilon\mathcal{L}_{\text{TopoLoss}}$ for 100 epochs using patches of 64 by 64, where $\epsilon = 0.2$.

As in VoxelMorph's implementation, ϵ was found to play a key role in performance, and therefore the value

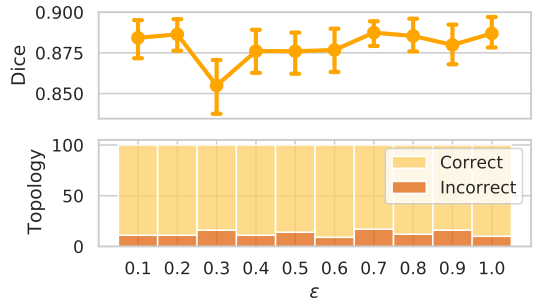

Figure A.2: The impact of the weighting factor, ϵ, between Dice overlap and TopoLoss in fine-tuning on the overlap segmentation performance.

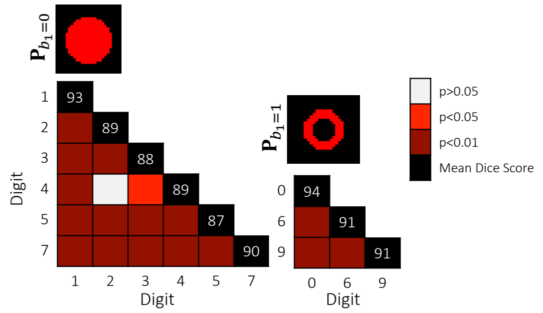

Figure B.3: A comparison of segmentation performance between digits segmented with the same prior. Significance (measured using an unpaired t-test and corrected with Bonferroni [2]) and average Dice (shown on the diagonal) is presented. Despite the same prior being used, the performance of the majority of the digits were found to differ significantly.

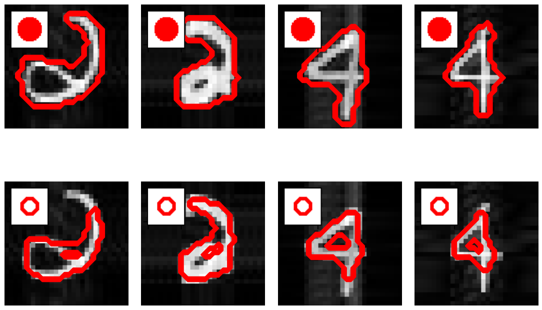

Figure B.4: Results obtained by replacing the prior shape used for 2 and 4 with $P_{b_1=1}$ at inference time. The top left corner of each image shows the prior used to produce the segmentation (shown in red).

of ϵ was varied to find the optimum performance, shown in Fig A.2. Due to the long runtimes of TopoLoss, shown in Table 1, for this experiment we only used 15% of the training set for fine-tuning. The best topology performance was found using $\epsilon = 0.2$, which was then used for the final experiment where we fine-tuned using 100% of the training dataset.

Following the authors' guidelines, post-processing operations were applied to the final predictions, as TopoLoss can create small disconnected components. We applied a binary opening operation using a kernel of size 3×3 to remove these isolated points, and it was found that this dramatically improved the topological performance.

Appendix B. Digit Segmentation

Natural variation in handwriting is particularly seen in digits *"2"* and *"4"*, where the digit can either be drawn as a single component with no hole or a component with a 2D-hole within it (e.g. *4* or 4). Therefore, depending on an individual's writing style, their topology can either be equivalent to $\mathbf{P}_{b_1=0}$ or $\mathbf{P}_{b_1=1}$. Initially, these digits were chosen to be segmented using $\mathbf{P}_{b_1=0}$, which often resulted in TEDS-Net surrounding the whole structure, and ignoring the hole, as shown in Fig. 7. To investigate the alternative topological profile of these digits, at inference time we replaced the prior $\mathbf{P}_{b_1=0}$ with $\mathbf{P}_{b_1=1}$, and the resulting segmentations are shown Fig. B.4. When changing the prior, the segmentation performance decreases, which is expected as the network has not learnt the fields based on the new prior. However, the predictions contain the equivalent topology to the new given prior. These results illustrate that the prior topology is still preserved, even in cases where the network has not seen a given prior and digit pair.

Appendix C. TEDS-Net Hyper-parameter Exploration

Appendix C.1. Interpolation Methods

Interpolation of the generated fields risks violating key topology-preserving properties, as discussed in Section 3.1. Across TEDS-Net, there are three instances where interpolation is applied: (i) field compositions layers, (ii) super up-sampling, and (iii) when resampling the prior. To explore the effect that the sampling had on topology-preservation, three interpolation methods, namely: linear, bicubic, and nearest neighbour, were used at each of these three instances, as shown in Fig. C.5.

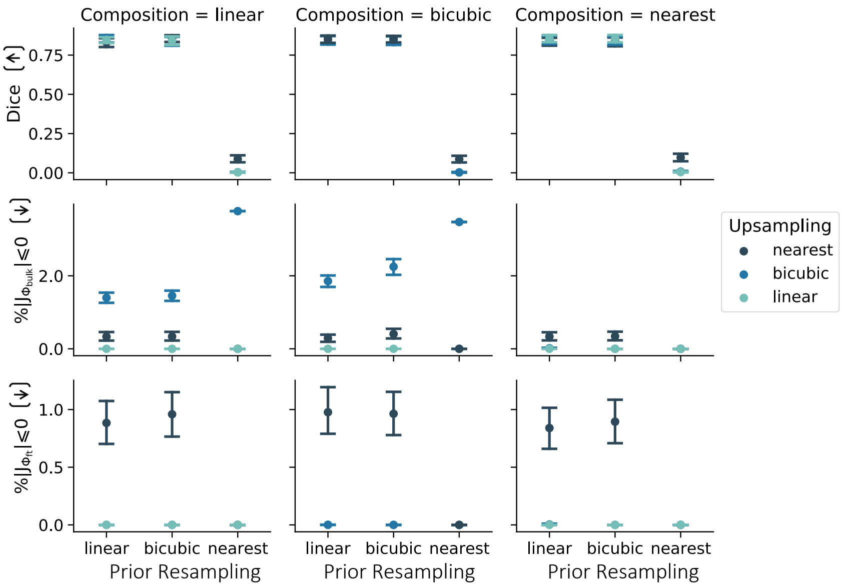

Figure C.5: Segmentation performance and percentage of folding voxels achieved under different interpolation methods. Interpolation is implemented at three instances in TEDS-Net, namely: (i) field compositions layers, (ii) super up-sampling, and (iii) when resampling the prior.

Across the combinations of interpolation methods, the worst image segmentation performance was found when sampling an image using *nearest neighbour* interpolation, regardless of the interpolation methods used for composition or upsampling. Using nearest neighbour for the final prior resampling yielded meaningless segmentations, resulting in a poor Dice performance. Nearest neighbour is known to lead to poor quality reconstructions, with block artefacts and lack of gradients, which is likely the cause of these results.

Sampling the prior shapes using either *linear* or *bicubic* interpolation results in similar segmentation performance across the different composition and upsampling methods. However, differences are seen in the number of folding voxels present in the fields. For instance, when using nearest neighbour for upsampling, folding voxels are introduced into both fields. This may be expected as the nature of this interpolation method can create discontinuities within an image. Using bicubic interpolation for the upsampling stage also introduced a small fraction of folding voxels within the bulk displacement field $\mathbf{\Phi}_{\text{Bulk}}$. Upsampling using linear interpolation was the only upsampling technique not to introduce folding voxels into the fields.

Setting the interpolation method for both the composition layer and prior resampling to either *linear* or *bicubic* resulted in strong segmentation performance and no folding voxels when used in combination with linear up-sampling. *Therefore, for the rest of the experiments we used linear sampling for all interpolations.*

Appendix C.2. Field Regularisation

Field regularisation loss functions are commonly used in registration formulations and spatial transformer implementations, to encourage smooth deformations [7, 1, 11]. Here, we investigate how adjusting the contribution of the two field regularisation loss weighting parameters, α and β, affects the nature of both the fields and the segmentation performance, shown in Fig. 9. As with varying sigma (shown in Fig. 9), the segmentation performance remains relatively unchanged across the range of weighting parameters, indicating that field smoothness does not strongly impact the pixel-wise segmentation performance.

Removing both the field regularisation terms ($\alpha = 0$, $\beta = 0$) results in folding voxels in both the bulk and fine-tuning fields, with a larger percentage found in the fine-tuning fields. This imbalance is likely to be caused by the additional smoothing applied to the bulk field when upsampling the fields. As the bulk field is initially at a lower resolution than the fine-tuning field, to upsample both to the same resolution (e.g. $2H \times 2W$), the bulk field undergoes more smoothing from the interpolation, which may result in fewer folded pixels within the field. Removing the fine-tuning field regularisation term ($\beta = 0$) and varying α, resulted in a handful of neg-

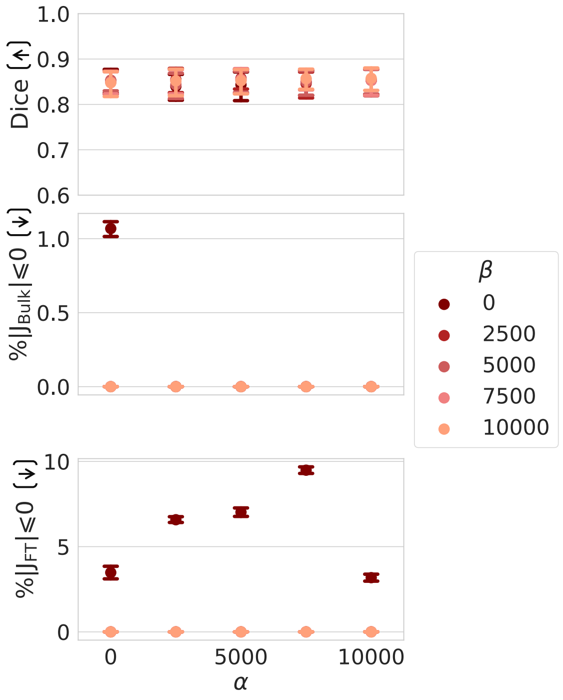

Figure C.6: Investigating how changing the weighting factors (α and β) within the field regularisation loss function affects the topology-preserving nature and segmentation performance of TEDS-Net. Note that, only $\beta = 0$ contained folding pixels, thus all other values of β are 0 in the bottom two plots.

ative Jacobian determinants within the fine-tuning field but not in the bulk field. This is also likely due to the additional smoothing in the bulk upsampling. As folding-voxels are present when no field regularisation is applied, despite the activation function and other smoothing modification (e.g. Gaussian smoothing) still in play, it suggests field regularisation is essential in enforcing topology-preserving fields within TEDS-Net.

For the rest of this work, we used balanced field regularisation weights of $\alpha = \beta = 10,000$.

In this work, we have chosen to regularise the fields by penalising local spatial variations in $\mathbf{\Phi}$ (shown in Equation 4) and by using appropriate weighting found this function was sufficient at removing folding voxels. However, alternative regularisation terms, such as the bending-energy [3], are available. Bending energy penalises the second derivatives of the fields, which in turn removes the affine contribution, returning smoother transformations than that shown in Equation 4. However, it is still less commonly used in the medical registration literature [6]. In future work, the effect of different field regularisation terms should be investigated, and whether they might the additional smoothing terms (e.g. the Gaussian Smoothing) redundant.

Appendix D. Domain Adaptation

The ACDC dataset was all collected from the same site (University Hospital of Dijon) using two Siemens MRI scanners. However, in practise, scans are acquired from a large range of sites and scanners, and the performance of segmentation models often decreases on unseen datasets. To investigate how well the different myocardium models coped under domain shifts, we evaluated each network on an unseen dataset. We used the open-source Multi-Centre, Multi-Vendor & Multi-Disease Cardiac Image Segmentation Challenge (M&M) [4], which is comprised of three vendors across four sites, and therefore offers a large domain shift from the ACDC training dataset. From the M&M dataset, the center slice was selected from each volume and then inspected to ensure the myocardium was visible, resulting in $N = 65$ labelled scans. Each slice underwent the same prepossessing as the ACDC dataset, as described above, before being applied to the trained networks.

Table D.1 shows the myocardium segmentation performance using the out of domain M&M dataset. The performance of each network decreased compared to the ACDC evaluation in Table 1, which is expected as the networks only saw ACDC images during training. TEDS-Net now outperforms Swin-Unet on Dice, however, both the U-Net and TopoLoss still achieve the best

Table D.1: Evaluation of the myocardium segmentation networks on unseen domain scans from the M&M dataset

Method	Dice ↑	HD ↓	Correct Topology ↑
U-Net	0.75 ± 0.18	30.80 ± 45.42	52%
Swin-Unet	0.66 ± 0.23	18.28 ± 22.72	50%
VoxelMorph	0.50 ± 0.29	33.88 ± 37.78	71%
TopoLoss	**0.80 ± 0.14**	16.76 ± 28.18	57%
TEDS-Net	0.71 ± 0.20	**15.50 ± 22.88**	**100%**

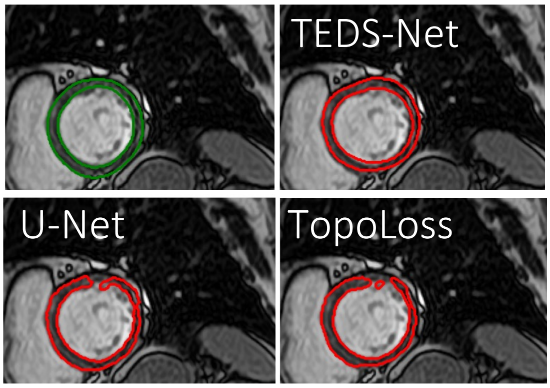

Figure D.7: An example domain shift myocardium segmentation from the top three performing networks. In this example, TopoLoss and the U-Net outperformed TEDS-Net on Dice, achieving 0.77 and 0.78, respectively, compared to TEDS-Net which achieved a Dice of 0.72. However, topological errors are present in the U-net and TopoLoss example.

Dice performance. Compared to the results in Table 1, the most stark change is on topology performance, where TEDS-Net still achieves 100%, with the next best performing network (VoxelMorph) only achieving 71%. Notably, TopoLoss's topology performance dropped from 95% to 57%. This implies that topology errors become more common in out of distribution data. Figure D.7, shows an example of a high quality segmentation from the TEDS-Net, TopoLoss and U-Net. In this example, TopoLoss and U-Net achieve a Dice score of 0.77 and 0.78, outperforming TEDS-Net's 0.72. However, both these networks have topological errors, which could make automated measures of perimeter or thickness challenging. Therefore, in this scenario, a 0.5 – 0.6% reduction in Dice but correct topology may be beneficial for downstream analysis tasks.

Appendix E. Myocardium Segmentation

Qualitative examples of myocardium segmentation across the networks are shown in Fig. E.8, Fig. E.9 and Fig. E.10, corresponding to the top, middle, and bottom five TEDS-Net segmentations in terms of Dice. On visual inspection, the segmentation quality across the baselines for the top (Fig. E.8) and middle (Fig. E.9) performing images seems consistent. However, when plotting Dice across the baselines for these images, as shown in Fig. E.11, a slight increase in performance is seen for U-Net, TopoLoss and Swin-Net, consistent with the findings in Table 1. Conversely, in Fig. E.10, the networks prediction heavily vary, which is reflected in Fig. E.11. Interestingly, for the worse TEDS-Net segmentations perform on par with TopoLoss.

References

[1] Balakrishnan, G., Zhao, A., Sabuncu, M. R., Guttag, J., and Dalca, A. V. (2019). Voxelmorph: a learning framework for deformable medical image registration. *IEEE transactions on medical imaging*, 38(8):1788–1800.

[2] Bonferroni, C. E. (1935). Il calcolo delle assicurazioni su gruppi di teste. *Studi in onore del professore salvatore ortu carboni*, pages 13–60.

[3] Bookstein, F. L. (1997). Landmark methods for forms without landmarks: morphometrics of group differences in outline shape. *Medical image analysis*, 1(3):225–243.

[4] Campello, V. M., Gkontra, P., Izquierdo, C., Martin-Isla, C., Sojoudi, A., Full, P. M., Maier-Hein, K., Zhang, Y., He, Z., Ma, J., et al. (2021). Multi-centre, multi-vendor and multi-disease cardiac segmentation: the m&ms challenge. *IEEE Transactions on Medical Imaging*, 40(12):3543–3554.

[5] Cao, H., Wang, Y., Chen, J., Jiang, D., Zhang, X., Tian, Q., and Wang, M. (2021). Swin-unet: Unet-like pure transformer for medical image segmentation. *arXiv preprint arXiv:2105.05537*.

[6] Chen, J., Liu, Y., Wei, S., Bian, Z., Subramanian, S., Carass, A., Prince, J. L., and Du, Y. (2023). A survey on deep learning in medical image registration: New technologies, uncertainty, evaluation metrics, and beyond. *arXiv preprint arXiv:2307.15615*.

[7] Dalca, A. V., Balakrishnan, G., Guttag, J., and Sabuncu, M. R. (2018). Unsupervised learning for fast probabilistic diffeomorphic registration. In *MICCAI*, pages 729–738. Springer.

[8] Dalca, A. V., Balakrishnan, G., Guttag, J., and Sabuncu, M. R. (2019). Unsupervised learning of probabilistic diffeomorphic registration for images and surfaces. *Medical image analysis*, 57:226–236.

[9] Guttag, J. and Dalca, A. V. (2021). Hypermorph: Amortized hyperparameter learning for image registration. In *Information Processing in Medical Imaging: 27th International Conference, IPMI 2021, Virtual Event, June 28–June 30, 2021, Proceedings*, volume 12729, page 3. Springer Nature.

[10] Hu, X., Li, F., Samaras, D., and Chen, C. (2019). Topology-preserving deep image segmentation. In *NeurIPS*, pages 5657–5668.

[11] McInerney, T. and Terzopoulos, D. (1996). Deformable models in medical image analysis: a survey. *Medical image analysis*, 1(2):91–108.

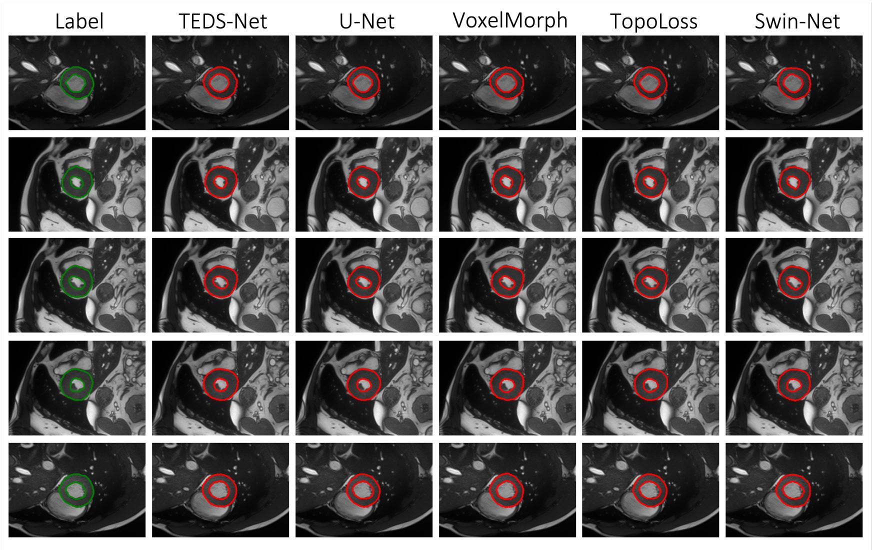

Figure E.8: The five best performing TEDS-Net segmentation in terms of Dice compared to the other baselines.

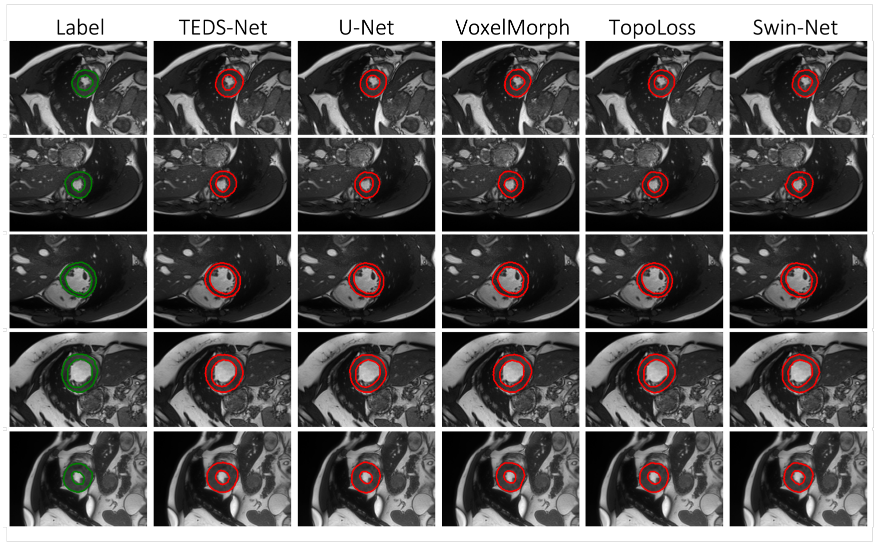

Figure E.9: The five mid-preforming TEDS-Net segmentation in terms of Dice compared, to the other baselines.

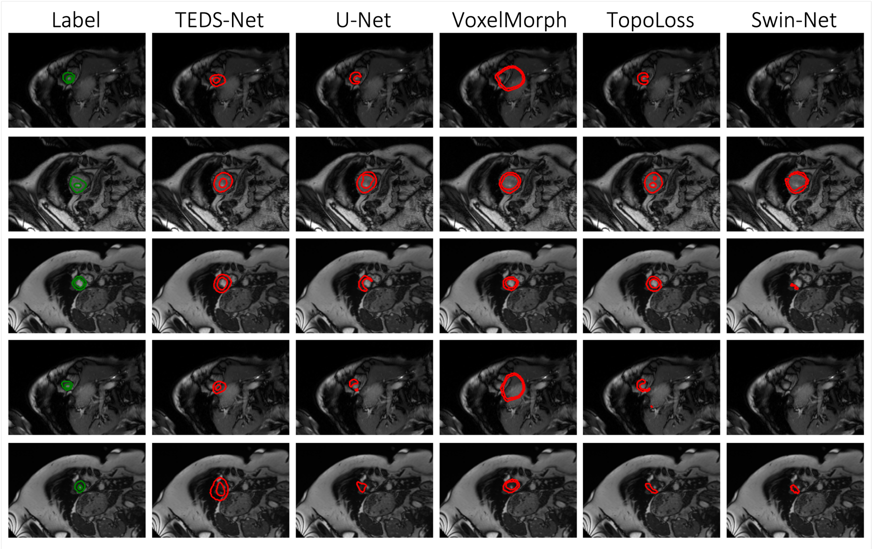

Figure E.10: The five worse performing TEDS-Net segmentations in terms of Dice, compared to the other baselines

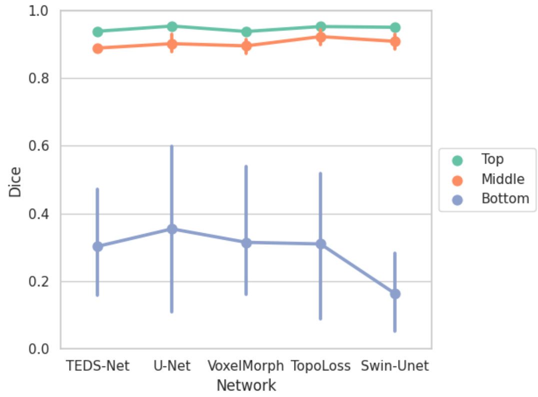

Figure E.11: The average Dice scores across the networks for the 3 groups displayed in Fig. E.8, Fig. E.9 and Fig. E.10.
